# Ferromagnetic Shape Memory Heusler Materials: Synthesis, Microstructure Characterization and Magnetostructural Properties

**DOI:** 10.3390/ma11060988

**Published:** 2018-06-11

**Authors:** Riaz Ahamed Ahamed Khan, Reza Ghomashchi, Zonghan Xie, Lei Chen

**Affiliations:** 1 School of Mechanical Engineering, University of Adelaide, Adelaide 5005, Australia; zonghan.xie@adelaide.edu.au (Z.X.); lei.chen@adelaide.edu.au (L.C.); 2 School of Engineering, Edith Cowan University, Joondalup WA 6027, Australia

**Keywords:** Heusler alloys, martensitic transformation, magnetic/metamagnetic shape memory, magnetocaloric, liquid and solid processing, microstructure

## Abstract

An overview of the processing, characterization and magnetostructural properties of ferromagnetic NiMnX (X = group IIIA–VA elements) Heusler alloys is presented. This type of alloy is multiferroic—exhibits more than one ferroic property—and is hence multifunctional. Examples of how different synthesis procedures influence the magnetostructural characteristics of these alloys are shown. Significant microstructural factors, such as the crystal structure, atomic ordering, volume of unit cell, grain size and others, which have a bearing on the properties, have been reviewed. An overriding factor is the composition which, through its tuning, affects the martensitic and magnetic transitions, the transformation temperatures, microstructures and, consequently, the magnetostructural effects.

## 1. Introduction

The term multiferroic refers to the simultaneous exhibit of multiple ferroic behaviors of ferromagnetism, ferroelectricity and ferroelasticity in materials. Each is characterized by the presence of a distinct hysteresis loop when switched magnetically, electrically or mechanically. Various factors, such as chemistry, symmetry, conductivity (conductor/insulator), mechanical distortion, etc., do not place any constraint on a material being multiferroic. Multiferroic behavior can therefore be engineered by bringing about a coexistence of phases with unlikely properties [1]. Several conditions, such as (a) phase boundary between phases, (b) phase transformation by application of fields or stresses, (c) sufficiently fast kinetics of transformation and (d) reversibility, are to be satisfied. Martensitic transformation in materials by virtue of being diffusionless could be a basis for the realization of multiferroic behavior. With reversibility and compatibility between phases ensured by tuning the composition, the ferroic orderings can be combined [1] with a large potential for applications. Heusler materials (e.g., Cu_2_MnAl: magnetic even though constituent elements are nonmagnetic, TiNiSn: semiconducting even though constituent elements are metals), discovered by Fritz Heusler in 1903, are multiferroic by martensitic phase transformation. Multiferroic effects in NiMnX (X = group IIIA–VA elements) Heusler materials [2] form the subject matter of this overview paper. Prominent effects seen in them are magnetic/metamagnetic shape memory (MSM/MMSM), magnetocaloric (MC) and direct energy conversion.

The terms MSM/MMSM are all-pervading in the discussion of these materials. Martensitic transformation renders variants in the product (martensite) phase, which possesses magnetic domains below the Curie point. Upon the application of a magnetic field and given a strong magnetic anisotropy of the material, the martensitic variants rotate to bring their easy axes in the direction of the applied field by utilizing the difference in their Zeeman energy levels in a manner similar to rearrangement by twin boundary motion under an applied stress [2]. Heating to above the austenite finish temperature reverts the product phase back to the parent phase and the large deformation from the application of magnetic field can be recovered. This is magnetic shape memory effect. Early research on magnetic field-induced strain (MFIS) in single crystals of Ni_2_MnGa alloys through twin boundary motion was reported in [3,4,5,6,7]. It has been found that necessary conditions for large MFIS are a low twinning stress (σtw) and a high energy of magnetic anisotropy [8]. The magnetic field-controlled strain response ε(MSM) is supposedly equal to the maximum strain ε_0_ = (1 − *c*/*a*, *c* and *a* are lattice parameters for tetragonal crystal structure) allowed by twinning when the magnetic anisotropy energy density Κ > ε_0_σ_tw_ (ε_0_σ_tw_—mechanical driving force) [8]. Metamagnetic shape memory effect, observed in off-stoichiometric Ga-free alloys, on the other hand, refers to the recoverability upon heating of large strains obtained when a reverse martensitic transformation from the product phase to parent phase is induced by the application of a magnetic field, with emphasis on the reverse transformation from martensite to austenite [9,10,11,12].

Magnetocaloric effect is given as the change in entropy induced by the applied magnetic field, measured by calorimetric measurements or from magnetization isotherms. Typically, it is quantified by an adiabatic temperature change or an isothermal entropy change occurring on the application or removal of an external magnetic field. The product of the changes in isothermal field-induced entropy (ΔS) and the adiabatic field-induced temperature (ΔT), |ΔSΔT|, is maximum when the magnetization change at the transition is maximum for an optimal composition in ferromagnetic Heusler alloys [13]. Interest in magnetocaloric materials was triggered by the discovery of a giant magnetocaloric effect (GMCE) in Gd_5_(Si_2_Ge_2_) around room temperature [14]. Research on MCE in NiMnX alloys followed later on, with the NiMnGa system most studied [15,16,17,18]. The observed large isothermal entropy change is broken up into the magnetic and structural entropy changes arising out of the spin-lattice coupling [2,19], occurring at the point where the martensitic transformation temperature and the magnetic transition temperature are close to one another [20]. A positive value of entropy change is termed as inverse effect and is related to the magnetocrystalline anisotropy of the martensitic phase. The inverse effect is more pronounced when the difference between the martensitic transition and Curie temperatures is an appreciable value. Conversely, a negative entropy change is termed conventional effect, and the change from inverse to conventional occurs as the martensitic transformation temperature approaches the Curie temperature at higher values of applied field [20,21]. Alloys such as Ni_0.50_Mn_0.50−x_Sn_x_, Ni_50_Mn_35_Sn_15_ and Ni_50_Mn_33.66_Cr_0.34_In_16_, which have been reported to exhibit inverse/conventional magnetocaloric/giant magnetocaloric effects, are reported in [10,22,23]. Polycrystalline Ni_45_Mn_43_CrSn_11_ alloy exhibited large inverse magnetocaloric effect with magnetic and total entropy changes of 35 J/kgK and 39.7 J/kgK, respectively [24].

The direct conversion of thermal energy (waste heat) to electricity is a more recent phenomenon, exhibited by a singular quaternary ferromagnetic Heusler alloy. A sudden and large thermally induced change in magnetization in a biased magnetic field initiated by phase transition was utilized to generate a voltage of 0.6 mV. This is using the fundamental dipolar relationship between magnetization M, magnetic induction (magnetic flux density) B and magnetic field H, given by B = H + 4πM and Faraday’s law $curlE=\frac{1}{c}\frac{\mathrm{dB}}{\mathrm{dt}}$ based on the premise that the magnetostructural transformation essentially induces a non-zero dB/dt [25]. This phenomenon, as envisaged by the researchers, could be taken to the stage of actual application, with the alloy having the potential to be used for harvesting energy from low waste heat sources of the order of less than 200 °C [25].

For all intents and purposes, the synthesis and microstructures play a vital role in the functionality of these materials. There are a large number of alloys prepared in this class of materials, evidenced by the number of publications appearing in ISI Web of Science each year [26]. Additionally, review articles have been published on MSM/MMSM materials [13] and magnetocaloric materials [2,20,27]. Particular to MCE, research is also being carried out on using materials with reduced dimensions, such as thin films, ribbons and microwires [28]. Even though there is a vast amount of literature about multiferroic materials proportionate to the growing interest in them, a review of how the synthesis procedures and microstructures influence their magnetostructural properties would be appropriate. This article mostly restricts itself to NiMn-based MSM/MMSM and MC materials, since it will be impossible to summarize the data of a large number of publications on a whole range of ferromagnetic alloy systems, most of which just report a new composition with an enhanced magnetostructural characteristic. This article is written with intent of providing enough information to the uninitiated researchers in order that they can meaningfully channelize their research and contribute their might toward extending the realm of these multiferroic materials from experimental to actual application.

The first section of this article deals with the fundamental concepts necessary for the understanding of the concept of multiferroic behavior in materials. The second section of this article deals with the fundamental concepts necessary for the understanding of the concept of multiferroic behavior in materials. The third section covers the synthesis and characterization procedures employed on these class of materials, with some inputs to design. The fourth section looks into how the magnetostructural characteristics of various ferromagnetic Heusler alloys are influenced by microstructural features.

## 2. Fundamental Concepts

### 2.1. Crystal Structures of Austenite and Martensite

The austenite structure in ferromagnetic Ni-Mn-X (X = Sn, Sb, In, Ga) alloy systems is of L2_1_ atomic order [2,29]. The cubic L2_1_ (space group F$\bar{4}$3m) structure has four interpenetrating face-centered cubic (fcc) sublattices. The crystal sites are designated as A (0, 0, 0), B (1/2, 1/2, 1/2), C (1/4, 1/4, 1/4) and D (3/4, 3/4, 3/4) in Wyckoff notations, which indicate positions of atoms in a crystal. Generally, in X_2_YZ Heusler alloys, the X atoms occupy (A, C) sites, Y atoms occupy B sites and the main group element Z occupies the D sites. Between X and Y, the one which has a higher number of valence electrons occupies the (A, C) sites and the one with fewer valence electrons occupies the B site. In some alloys, an order–disorder phase transition (L2_1_–B2) occurs, transforming the nonequilibrium B2 to a more stable L2_1_ phase. This sometimes causes confusion in distinguishing the two phases correctly from electron diffraction studies, as the atomic scattering is similar. The B2′ structure has Y/Z atoms occupying A positions and X atoms occupying B positions and exhibits a first neighbor ordering, while L2_1_ exhibits a second neighbor ordering [30]. The austenitic structure is L2_1_ at room temperature in the case of quaternary Heusler alloys with the stoichiometry defined by 1:1:1:1. Wyckoff atomic positions 4a (0, 0, 0), 4b (1/2, 1/2, 1/2), 4c (1/4, 1/4, 1/4) and 4d (3/4, 3/4, 3/4) are occupied by Z, Y, X and X’, respectively, in XX’YZ alloys (X = Ni, X’ = Co, Y = Mn and Z = main group element) [31]. As in ternary alloys, in quaternary alloys, B2-type structural disorder appears with variation of the Z element, particularly Al.

**Figure 1:**
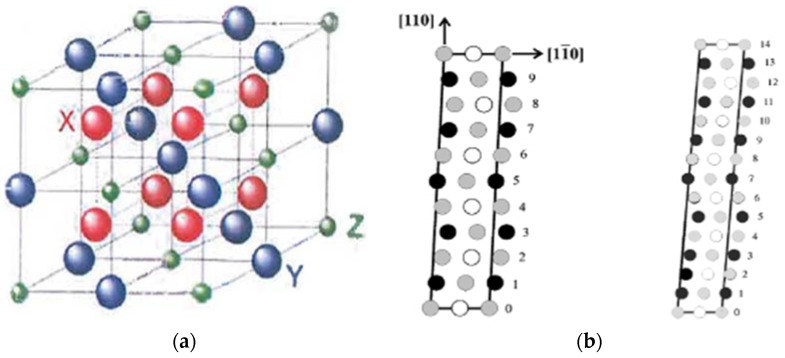
(**a**) The L2_1_ structure representing the crystalline structure of austenite [32], with copyright permission from © Trans Tech Publications; (**b**) the 5*M* and 7*M* modulated structures of martensite. Light grey—X, white—Y, black—Z [2], with copyright permission from © IOP Publishing.

The martensite structure varies from a body-centered tetragonal (with *c*/*a* < 1) and is modulated along the 〈100〉 crystallographic direction of L2_1_ lattice for stoichiometric alloys to layered 5*M* or 7*M* structures, or even unmodulated body-centered tetragonal (L1_0_, with *c*/*a* > 1) for compositions different from stoichiometric. The martensite structure also varies with the dopant and its concentration. It is interesting to note that depending on the temperature of martensitic transformation M_s_, the martensite is five-layered for both stoichiometric and off-stoichiometric compositions below M_s_, five- or seven-layered near M_s_ and seven-layered/10*M*/unmodulated above M_s_ [30]. The L2_1_ structure of austenite [32] is shown in Figure 1a. The 5*M* (also referred to as 10*M*) and 7*M* (also referred to as 14*M*) modulated structures of martensite are shown in Figure 1b. Martensitic structures can even coexist in the same composition, e.g., 6*M* and 10*M* [9] and 10*M* and 14*M* [33]. The different stacking sequences of the martensite are evaluated on the basis of the 2*M* structure, which has a unique lattice correspondence with the L1_0_ structure [34].

**Figure 2:**
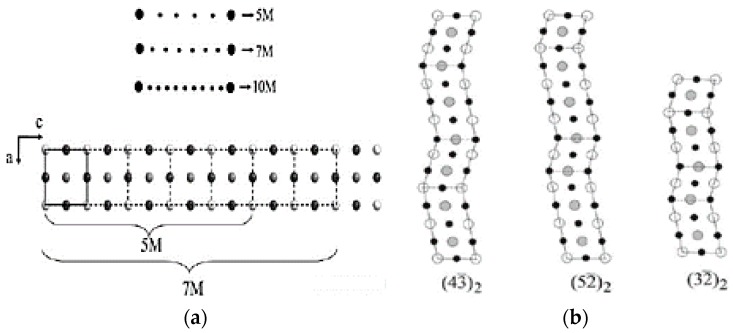
(**a**) Graphical representation of the nM = s + 1 relationship; (**b**) three examples of Zhdanov sequences of martensitic layered surfaces—7*M* and 5*M* modulations [35], with copyright permission from © Trans Tech.

The structural modulations of the martensite are periodic stacking faults of atomic planes along determined crystallographic directions, seen as extra reflections in XRD and TEM characterization [35]. These reflections enable the modulations to be represented as nM, indicating n-fold modulation, where n = s + 1 (‘s’ being the satellite spots lying between main spots) is the number of unit cells that constitute the superstructure, as in 5*M*, 6*M*, 7*M*, 10*M* and 12*M* martensite with 5, 6, 7, 10 and 12 unit cells, seen in Figure 2a. On the other hand, the Zhdanov notation identifies the modulation of martensitic structures as a series of numbers indicating the atomic layers in a periodic shift corresponding to the fundamental lattice vector, with the minus sign indicating the opposite shift and the suffix indicating the number of zigzag motifs which make up the crystal lattice, shown in Figure 2b. However, the incommensurateness associated with the structure is best described by the (3 + 1) superspace approach. An additional supplementary vector (modulation vector) is used to index the weak diffraction peaks seen in the diffraction data. The generic diffraction vector ***H*** becomes

H=ha*+kb*+lc*+mq

where q=αa*+βb*+γc*.

Each Bragg reflection has four indices *hklm* corresponding to a*, b*, c* (conventional lattice) and ***q***. When coefficients α, β and γ correspond to a rational number, the modulation is commensurate, and when they correspond to an irrational number, the modulation is incommensurate, requiring the introduction of superspace [35].

### 2.2. Magnetostructural Coupling and Magnetic Behavior

The mechanism of magnetostructural coupling in Heusler materials is very startling. Physicists and materials scientists have used density functional theory (DFT), ab initio methods and several approximations of DFT, such as the Perdew, Burke and Ernzerhof (PBE) functional, to understand magnetostructural coupling and determine the underlying principles which govern it. The magnetic ordering is reported to occur through an indirect oscillatory Ruderman–Kittel–Kasuya–Yosida (RKKY) exchange interaction [13,19], since there is no direct overlap between neighboring magnetic electrons. This magnetic ordering induces a hybridization of the electronic states through band Jahn–Teller (J–T) effect [36,37], which influences the martensitic transformation. In Heusler alloys, the magnetic moments are localized at the *d* states of Mn atoms. These localized moments overlap to cause ferromagnetic ordering. However, the shortest distance between Mn–Mn atoms is ~4.2 Å, which is insufficient for a direct overlap of the localized moments. Therefore, spin polarization of conduction electrons induced by the localized moments indirectly link the actual localized moments to create either a ferromagnetic/antiferromagnetic order [13]. Mn–Mn pairs at 4a (0, 0, 0) positions couple ferromagnetically and close neighbors Mn–Mn pairs at 4a–4b (0, 0, 0–1/2, 1/2, 1/2) positions couple antiferromagnetically. The martensitic transformation itself is known to occur in Heusler alloys at off-stoichiometric compositions. The magnetic moments from local distortions evident from shorter Ni–Mn bond distances cause hybridization between the Mn(d) and Ni(d) states at or near Fermi level and influence the martensitic transformation. In NiMnGa alloys, the hybridization is between spin-down 3*d* electrons of Ni and 4*p* electrons of Ga.

The magnetic order state of martensite is paramagnetic, mixed ferro-antiferromagnetic or even ferromagnetic. At lower temperatures, ferro- and antiferromagnetic components show up, leading to a complex state that is dependent on the doping element. Cong et al. reported martensite as paramagnetic, superparamagnetic (SPM) and superspin glass (SSG) in different temperature ranges during cooling [38] for compositions 0 ≤ *x* ≤ 8 in the Ni_50−x_Co_x_Mn_39_Sn_11_ alloy system. SPM refers to the magnetic behavior with no magnetic hysteresis (zero remanence and coercivity). Its M(H) curve fits the Langevin model given as

$$M\left(H\right)=N\bar{\mu }L\left(\xi \right)+\chi_{0}H$$

where $L\left(\xi \right)=\coth\left(\xi \right)-\frac{1}{\xi }$ is the Langevin function, $\;\xi =\mu_{0}\bar{\mu }H/k_{B}T$, $\chi_{0}$ is the magnetic susceptibility, $\;\bar{\mu }$ is the average magnetic moment, $\;\mu_{0}\;$is the vacuum permeability, *N* is the cluster density of SPM clusters and $k_{B}$ is the Boltzmann constant. SSG has a spin-configuration which is random in nature, similar to a paramagnet frozen in time [39]. SPM and SSG behaviors are understood from magnetic susceptibility measurements at lower temperatures and at different frequencies. SSG behavior is further explored from aging, rejuvenation and memory experiments conducted on a magnetometer. More information on the details of the experiments can be read in [38]. Additionally, the spin glass state occurs when the crystallite size is less than a critical value of 10 nm, with its morphology comprising a combination of small grains (<10 nm) and nanograins (~100 nm), as seen in electrodeposited Ni–Fe permalloy films [40,41]. Magnetic configurations in quaternary alloys are in accordance with the occupancy of the fourth element [42].

**Figure 3:**
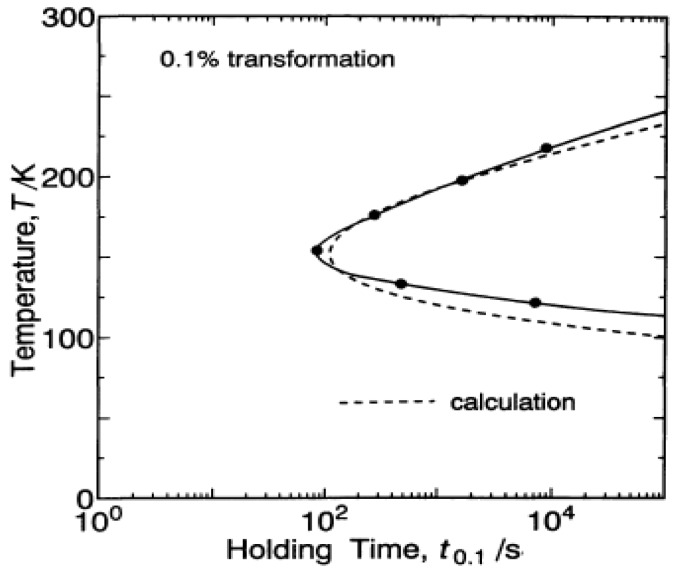
Time-temperature-transformation (TTT) diagram of the isothermal martensitic transformation in Fe-Ni-Mn alloy. Thick lines with closed circles represent measured values and dotted lines represent calculated values [45], with copyright permission from © JIM.

The formation of SSG or magnetic glass state is linked to isothermal transformation, wherein the austenite–martensite transformation is arrested when held isothermally in high magnetic fields. The knowledge of the kinetics of transformation becomes therefore necessary. Based on transformation kinetics, martensitic transformation is classified as either isothermal or athermal [43,44]. While in the former the amount of martensite formed depends on both time and temperature, in the latter it is dependent only on temperature, with both transformations influencing the electronic state of ferromagnetic Heusler alloys [45]. The description of athermal transformation is contrary to thermally activated transitions, wherein the relaxation from a metastable state occurs due to thermal fluctuations. Attention is drawn to the fact that martensitic transformation occurs through a cooperative movement of a large number of atoms at intersonic speeds (speeds exceeding the materials shear wave velocity), implied from a report on twin motion being faster than the speed of sound [46]. The compositions of the product and parent phases are the same, which serves to exclude time-dependence of the transformation. The kinetics changes from isothermal to athermal in the presence of high magnetic fields [47]. From an analysis of Fe-Ni-Mn alloys, Kakeshita et al. proposed a model to explain the natures of both transformations [45]. Assuming that martensitic transformation may start when a minimum size of a cluster of particles is formed and simultaneously excited in the austenite, the model accounts for the presence or absence of a C-curve in the time-temperature-transformation (TTT) diagrams of isothermal and athermal transformations, respectively, by considering the temperature dependence of Gibbs chemical free energies, Δ*G(T)*, of austenite and martensite (see Figure 3), in which isothermal transformation of the alloy formed an asymmetric C-curve, with the time required to form 0.1% volume fraction of martensite being different on the higher and lower temperature sides. Lee et al. also confirmed the formation of a C-curve in the TTT diagram of an Ni_45_Co_5_Mn_36.5_In_13.5_ alloy [48].

Several studies on isothermal/athermal martensitic transformation have been reported [49,50]. The kinetic arrest of martensitic transformation under a magnetic field during cooling and the subsequent increase in the amount of martensite phase during heating under zero magnetic field is attributed to the low mobility of the habit plane between austenite and martensite phases [51]. Thermomagnetization, electrical resistivity and X-ray structural analysis studies confirm the kinetic arrest phenomenon (incomplete transformation), in which the kinetics of the martensitic transition (first order) gets disrupted. The resulting low-temperature phase, which is not in an equilibrium state, has both fractions of the transformed stable martensite and metastable high-temperature austenite phases. The metastable state is referred to as magnetic glass, with the applied magnetic field either increasing or decreasing the kinetic arrest. This leads to the condition that when the alloy is cooled and heated in differing magnetic fields (H_C_ and H_W_) [52], the de-arrest or unfreezing of magnetic glass happens and a re-entrant transition (reverse magnetic transition) happens for a critical value of (H_C_ and H_W_).

## 3. Methods of Synthesis and Characterization

It is pertinent that the design aspects of an alloy system are known. Different ways of design are discussed which are central to synthesis and characterization of ferromagnetic Heusler alloys. An important characteristic of ferromagnetic Heusler materials is the reversibility of martensitic transformation that holds the key to the realization of coexistent phases with different electromagnetic properties [1]. Moreover, as the transformations are composition-dependent, tailoring the composition suitably will enhance the reversibility of the martensitic transformation such that switching between coexistent phases (austenite and martensite) occurs cyclically without any diminishing of properties, electromagnetic in particular. Mathematical conditions called cofactor conditions, adapted from the geometric nonlinear theory of martensite (GNLTM), provide a definite way to design highly functional alloys [53]. The geometrical conditions satisfy the premise that there should be no stressed transition layer between switching phases for the realization of least thermal hysteresis and enhanced reversibility.

The GNLTM solutions, as such, correspond to the twinning volume fractions, *f* and 1 − *f* [53]. When *f* = 0 or *f* = 1, the absence of an elastic transition layer between the austenite and the single variant martensite interface is possible when the middle eigenvalue λ_2_ of a transformation stretch matrix ‘U’ (3 × 3, obtained from X-ray measurements of lattice parameters and knowledge of space groups of the two phases) takes a value 1. For other volume fractions given by 0≤f≤1, two additional conditions corresponding to different twin types are to be satisfied. The volume fraction of the twin variants can then be continuously varied, while at the same time keeping the low-elastic-energy interface with austenite [53]. Using these conditions on the basic Ni_50_Mn_50−x_Sn_x_ alloy system, a composition Ni_45_Co_5_Mn_40_Sn_10_ was perfected with unusual magnetostructural properties [54]. The efficacy of the compatibility conditions have been proved experimentally in Zn-Au-Cu [53], Ti-Ni-Pd [55] alloys and used in modeling the austenite–martensite interface in shape memory materials [56].

Another approach to synthesis of ferromagnetic Heusler materials is by the combinatorial approach, which accelerates the discovery and optimization of known and new materials by combining efficient synthesis of a large number of different material compositions and high-throughput property screening methods to delineate composition–structure–property relationships and hence identify compositions with desired properties [57]. Combinatorial materials synthesis combined with GNLTM will certainly help in accelerated discovery of new and highly multifunctional Heusler alloys. More about combinatorial materials synthesis is detailed in Section 3.1.

**Figure 4:**
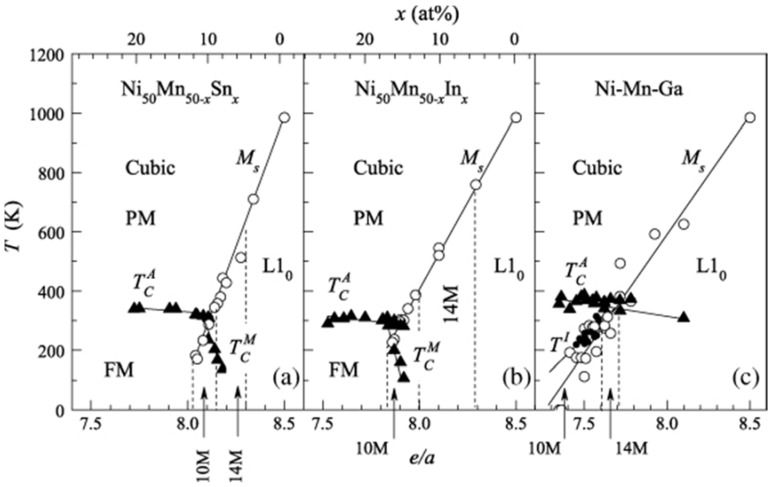
The magnetic and structural phase diagram of Ni-Mn-Z Heusler alloys with Z as: (**a**) Sn; (**b**) In; (**c**) Ga. The triangles and circles correspond to the magnetic and martensitic transformation temperatures, respectively. The regions corresponding to the different structures are separated by discontinuous lines [2], with copyright permission from © IOP Publishing.

It is possible to predict compositions having room temperature martensitic transformations or martensitic structures through the determination of the valence electron concentration per atom, written as the *e*/*a* ratio. An *e*/*a* value of 7.6 favored room temperature martensitic transformations [58]. The structure changed from cubic–10*M*–14*M*–L1_0_ in accordance with an increasing *e*/*a* ratio and temperature [2], as shown in Figure 4.

The *e*/*a* ratio is calculated as concentration-weighted average of the valence (s, p and d) electrons using the expression

ea= fNieNi+ fMneMn+ fXeX+fZeZ

where $f_{Ni}$, $f_{Mn}$, $f_{X}$, $f_{Z}$ represent the atomic fractions of the elements, $e^{Ni},\;e^{Mn}\;,\;e^{X}\;,\;e^{Z}$ are the corresponding numbers of valence electrons and, X, Z represent the third and fourth elements of a quaternary alloy, respectively.

Phase equilibria information about Ni–Mn-based alloys, which would equip researchers in alloy development, is scarce. The work by Yang et al. in consolidating the phase equilibria of the Ni-Mn-Ga system, using the equilibrium compositions obtained from the diffusion couples and two-phase alloys, highlights critical compositions favoring near room temperature martensitic transformations as those with *e*/*a* of 7.6 [58]. A similar work on Ni-Mn-In alloy system confirmed a single β phase region existing over a composition range of 0–20 atom % In, in addition to the martensite [59]. Microstructure observation with magnetic colloid (Magnetic colloid refers to a technique of observation of magnetic structures smaller than 100 nm. Ferrofluids with magnetic particles like Fe_3_O_4_, γ-Fe_2_O_3_ or metallic particles of iron, nickel or cobalt having 10–15 nm diameter dispersed in water or an inorganic liquid are used for imaging the fields of magnetization. The particles in the ferrofluid interact by magnetic forces and by electrostatic and van der Waals forces. The liquid is usually dried up or rinsed, leaving the colloid particles to form Bitter patterns, after F. Bitter) [60] yielded a critical boundary between the ferromagnetic and paramagnetic phases spread over a wide composition range from the Ni-rich region to the Mn-rich region.

**Figure 5:**
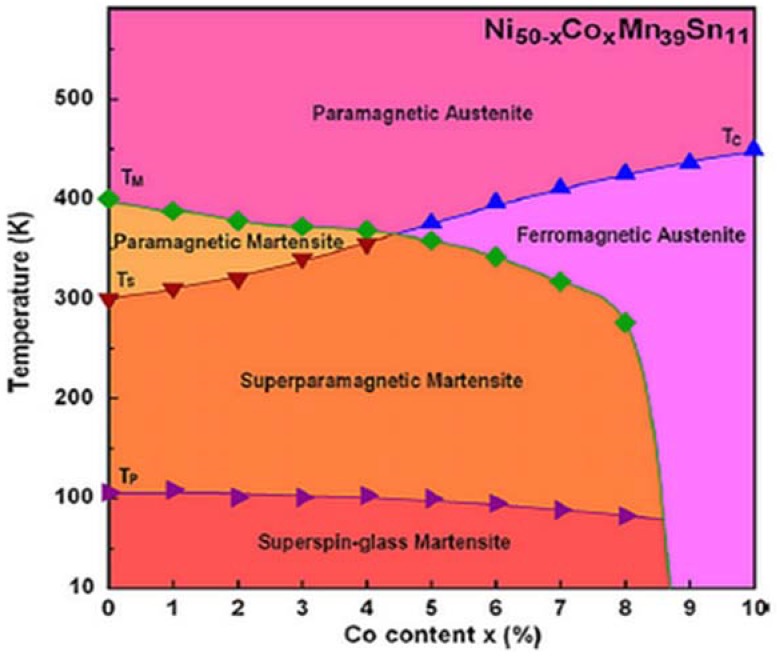
Phase diagram of Ni_50−x_Co_x_Mn_39_Sn_11_ (0 ≤ *x* ≤ 10) alloy system [38], with copyright permission from © Elsevier.

However an isothermal section of the Ni-Mn-In ternary phase diagram at 773 K showed a host of single, two- and three-phase regions [61] with no bearing on the martensitic and magnetic behavior. A vertical section of the Ni_50_Mn_50−x_Sn_x_ system (0 ≤ *x* ≤ 50 atom %) drawn from magnetic susceptibility measurements and saturation magnetization vs. temperature, structural and X-ray investigations also identifies different low- and high-temperature phases as also a face-centered cubic γ phase at small Sn concentrations [62]. Martensitic structures formed after annealing at high temperatures followed by quenching in water for Sn content from 0 to 11 atom %. Phase diagrams showing both high- and low-temperature regions of Ni_50−x_Co_x_Mn_39_Sn_11_ quaternary Heusler system enhance the understanding of the composition and temperature-dependent functional properties and associated physical phenomena [38], shown in Figure 5.

### 3.1. Synthesis

Ferromagnetic Heusler materials are synthesized through both liquid and solid processing routes. The commonly employed processing route is melting and casting [63]. The techniques include arc/induction melting under controlled conditions and rapid solidification by melt spinning. Directional solidification using Bridgman–Stockbarger [64] and Czochralski [65] techniques also have been used. Solid processing is by powder metallurgy (P/M) using alloy [66] and elemental [67] powders. Sintering techniques include pressureless sintering [68,69], spark plasma [70] and solid-state replication to obtain a porous alloy sample [71].

Liquid processing route has been predominantly applied in synthesizing the Heusler alloys primarily because of the compositional homogeneity obtained after several rounds of remelting. The alloys are melted, turned over and remelted in arc or induction melting furnaces, either in vacuum or an inert atmosphere. The arc melting furnace uses an electric arc struck between an electrode (tungsten) and the metal mix placed in crucibles for melting where temperatures in excess of 2500 °C can be reached. Induction melting, on the other hand, combines the advantages of a controlled atmosphere and control of the melting process, where heating is by eddy currents from an induction coil with no direct contact between the metal and coil. The process capability is defined by temperatures of up to 2000 °C that can be reached [72].

**Figure 6:**
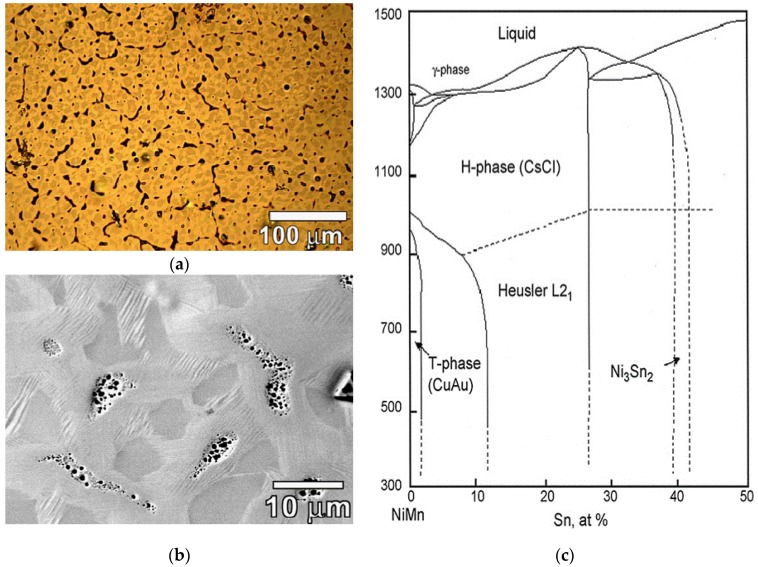
(**a**) Optical micrograph of as-cast Ni_44.5_Co_5.5_Mn_39.5_Sn_10.5_ alloy [73], with copyright permission from © Elsevier; (**b**) SEM micrograph of the as-cast alloy [73], with copyright permission from © Elsevier; (**c**) vertical section of phase diagram [62], with copyright permission from © Elsevier BV.

Liquid processing yielded multiphase microstructures, as can be seen from the investigations by several researchers. L2_1_/martensite, DO_3_ and γ (disordered fcc) phases were observed in the as-cast microstructures of various Ni-Co-Mn-Sn alloys [73], shown in Figure 6a,b. Cong et al. reported the presence of unidentified second-phase particles, even after annealing in quaternary Ni_50−x_Co_x_Mn_39_Sn_11_ alloy system for *x* = 9 *x* = 10 [73]. Ni-Mn-Sn alloys solidified as multiphase microstructures, which affected both the phase stability of the L2_1_ phase and martensitic transformation [74]. Cubic DO_3_, L2_1_ and hexagonal DO_19_ phases were reported in Ni_75−x_Mn_x_Sn_25_ (0≤x≤40) alloys [75]. The multiphase microstructures, however, changed to single-phase microstructures upon annealing. The vertical section of the phase diagram of the Ni_50_Mn_50−x_Sn_x_ (0≤x≤50) alloy system in Figure 6c shows the as-solidified alloy changing to a single-phase structure [62]. If the compositional variations are still seen, it is because chemical variations require longer annealing treatments than the time scale for structural conversion.

**Figure 7:**
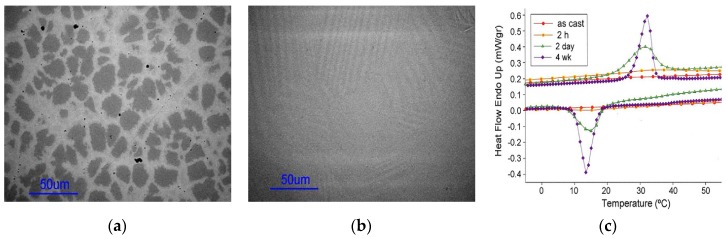
(**a**) As-cast; (**b**) heat-treated for 72 h; (**c**) differential scanning calorimetry (DSC) data showing the effect of heat treatment [76], with copyright permission from © Elsevier BV.

Since phase transformations in ferromagnetic Heusler alloys are highly composition-dependent, it is necessary that chemical variations are equilibrated over extended time scales through long annealing treatments and not limited to shorter time scales used for structural conversion in order that compositional homogeneity is achieved [76]. The magnetic and structural transitions then become distinct. Figure 7a shows the as-cast structure of Ni_50_Mn_37_Sn_13_ ternary alloy which exhibited multiphase microstructure, which changed to single-phase microstructure rapidly upon annealing. Figure 7b shows of the sample annealed at 950 °C for 72 h, during which the compositional variations were eliminated. Extended annealing periods of up to 4 weeks sharpened the thermal peak of martensitic transformation, shown in Figure 7c, which was a result of homogenization occurring through the duration of annealing [76].

**Figure 8:**
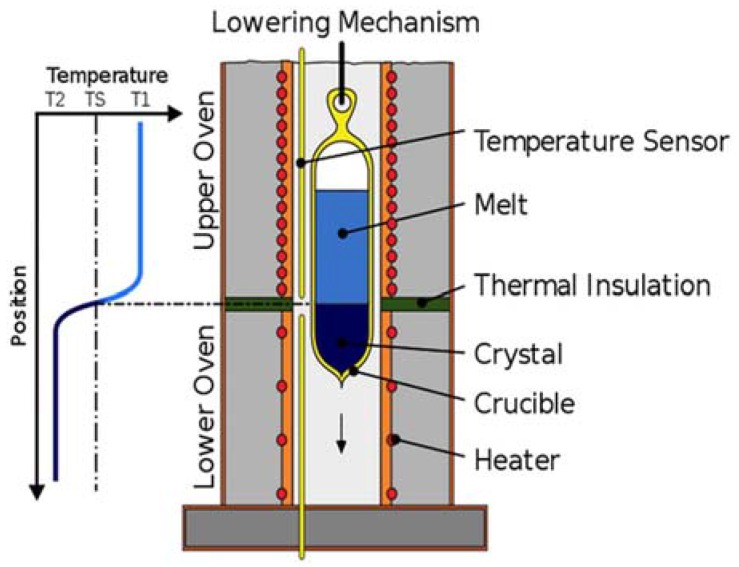
Schematic of the Bridgman–Stockbarger method [77].

Another dimension to synthesis of polycrystalline magnetic shape memory alloys is the application of directional solidification to prepare highly oriented alloys [64]. These alloys exhibit a large pseudoelastic recovery besides chemical segregation, or a composition gradient due to which changes in transformation temperatures are obtainable. Czochralski and Bridgman–Stockbarger techniques are the two widely employed techniques of directional solidification for synthesizing ferromagnetic shape memory alloys. The Bridgman–Stockbarger method has a high-temperature zone, an adiabatic loss zone and a low-temperature zone. While Czochralski technique is known for its relatively high growth rate, modified Bridgman–Stockbarger technique employs gradient freezing, which requires no translation of either the crucible or furnace, and temperature gradient is affected through programmed control of the multiple heat zones in the furnace. A schematic of the Bridgman–Stockbarger method [77] is shown in Figure 8.

**Figure 9:**
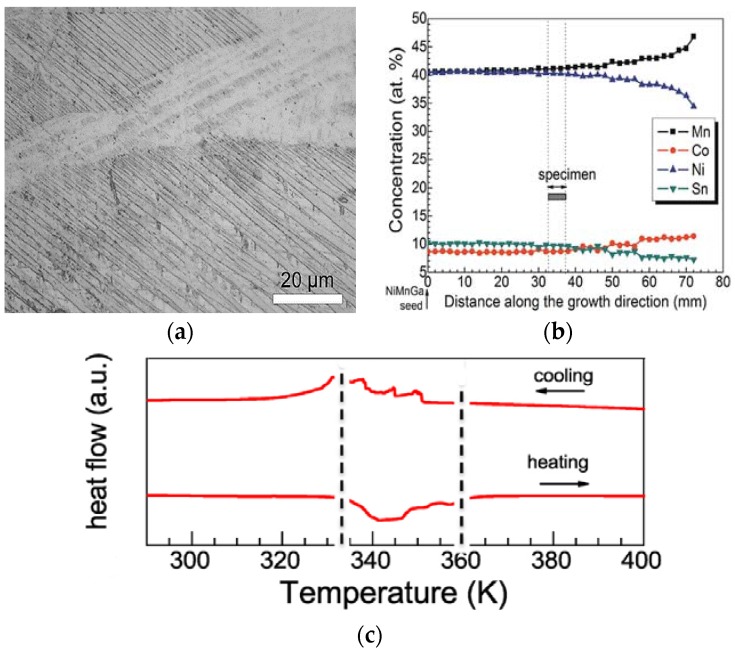
(**a**) Microstructure of directionally solidified alloy; (**b**) composition profile along the growth direction; (**c**) DSC curves across the structural transition [64], with copyright permission from © Elsevier BV.

A unidirectional crystal of Ni_40.6_Co_8.5_Mn_40.9_Sn_10_ grown using Bridgman–Stockbarger technique [64] yielded a microstructure which had no secondary γ phase precipitate, as seen in Figure 9a. The resulting compositional segregation with an increase in Ni, Sn and decrease in Mn, Co, as shown in Figure 9b, induced a steplike martensitic transformation, shown in Figure 9c, which broadened the working temperature range for magnetic entropy [64]. The magnetic entropy change and refrigeration capacity were better than those in Ni_40_Co_10_Mn_40_Sn_10_ [78], Gd_5_Ge_2_Si_2_ [79] and Ni_50_Mn_36_Sn_14_ [80] alloys, reported for their exceptional magnetocaloric properties. The functional properties of magnetic shape memory alloys are usually best seen in single crystals, as in [81].

Other synthesis methods which have been used on ferromagnetic shape memory alloys are hot-forging and rolling, where the resultant strong textures and large in-plane plastic flow anisotropy are attributed to the rearrangement of martensitic variants during the thermomechanical processes [82,83,84]. The Taylor–Ulitovsky method of fabricating glass-coated microwires [85] is increasingly being applied on MCE materials [86,87,88,89,90]. The process, which has a capability of up to 10,000 m of continuous microwire, consists of induction heating a few grams of the master alloy in a borosilicate glass tube. As the alloy is melted, the glass tube softens around the molten alloy droplet, from which a glass capillary is drawn out with the alloy filling the capillary and forming a microwire. The microwire has the glass shell surrounding the alloy (metal) core. The formation of the core is limited by the initial amount of master alloy and its microstructure is dependent on the rate of cooling [85] as it is wound on a receiving coil. The microstructure of as-cast microwire of Ni_49.5_Mn_25.4_Ga_25.1_ was seen to consist of two phases—tetragonal I4/mmm and cubic Fm3m [86], which, after annealing, turned into single phase favoring martensitic transformation.

Rapid solidification processing with solidification rates ranging from 10^2^ K·s^−1^ to 10^14^ K·s^−1^ [91] has been a very useful method for preparing ferromagnetic Heusler alloys. The characteristics of rapid solidification processing are extension of solid solubility limits, microstructural refinement and formation of nonequilibrium phases, such as metastable intermediate phases, metallic glasses and quasicrystalline phases with crystallographically forbidden 5-fold, 10-fold and other symmetries [91]. The discussion about crystallographically forbidden lattices is beyond the scope of this overview, even though such microstructures are not reported in Ni–Mn-based Heusler alloys as they transform to modulated, commensurate/incommensurate 6*M*, 10*M*, 14*M*, 4*O* or L1_0_ martensitic structures. Crystallographically forbidden lattices in conventional shape memory materials, such as Cu-Al-Ni [92] and Cu-Zn-Al [93] alloys, modify the normal 2*H* (orthorhombic) microstructure of the martensite.

Melt-spinning as a rapid solidification technique is gaining wide acceptance in synthesizing Heusler alloys [94,95,96] for varying reasons, such as obtaining textured samples suitable for practical utilization as sensors, actuators and magnetocaloric materials which optimize the heat transfer in a refrigeration unit [97,98,99]. Furthermore, it lends credence to the fact that the processing methods can tailor the functional attributes of Heusler alloys by controlling the lattice parameters, interatomic distance, degree of atomic ordering and microstructure. It is primarily employed to control the grain size of the austenite phase. This is because the martensitic transition can occur only when the austenite grain size is bigger than the martensite plate [100]. Also, homogeneity in terms of grain size and elemental composition is achieved by melt spinning [100]. Another reason for using melt spinning is that it favors avoidance of prolonged annealing.

Synthesis by melt spinning is by allowing the alloy melt stream jet to solidify rapidly on a fast-rotating and thermally conducting substrate to produce a continuous strip or ribbon of the alloy up to 500 mm in width [91]. Wheel speed, nozzle size, ejection pressure and material of the rotating substrate (wheel) are some of the parameters which influence the process. Typical wheel speeds vary from 10 m/s to 60 m/s and the substrate is usually copper. Melt spinning has the effect of eliminating the secondary γ phase in Ni_38_Co_12_Mn_41_Sn_19_ alloy as a result of which an enhancement of magnetocaloric properties equivalent to the bulk alloy was observed [101]. The effective refrigeration capacity (RC_eff_) values of ~48.8 and 47.8 J/kg were obtained for as-spun ribbons and annealed ribbons prepared at 15 m/s and 25 m/s, respectively [99]. The MCE characteristics of as-cast and annealed melt-spun Ni_50_Mn_35_In_14.5_B_0.5_ alloy ribbons, in which the isoelectronic B is substituted for In, were identical to the bulk alloy. A relative cooling power of 150 J/kg for annealed ribbons was reported [102].

Solid processing using P/M has also been applied to synthesize both conventional [103,104,105,106,107,108] and ferromagnetic shape memory materials [66,67,68,69,70,71], although its application has been widespread in the former category. Apart from the application of P/M in conventional shape memory materials, its observed benefits have been the ability to obtain the desired martensitic transformation temperatures, transformation width (range) and hysteresis, as in Cu-Al-Ni [109], drop in transformation temperatures, as in Ti-Ni-Cu [110] and, more importantly, significant reduction in the number of secondary phases using vapor phase calciothermic reduction (VPCR) in solid-state sintering of Ni_50_Ti_50−x_Zr_x_ [111].

**Figure 10:**
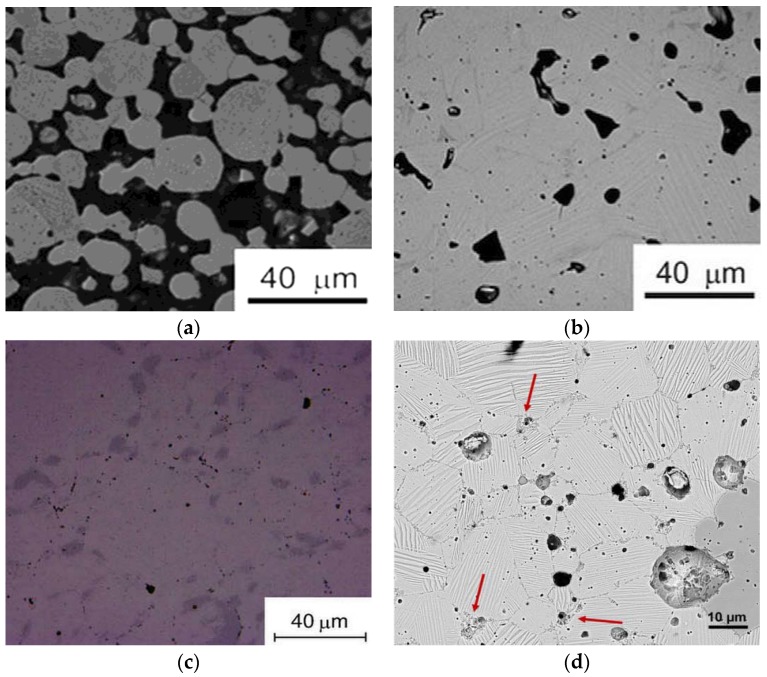
Porous sample sintered for: (**a**) 12 h; (**b**) 144 h [68], with copyright permission from © Pergamon; (**c**) sample synthesized by spark plasma sintering [70], with copyright permission from © Pergamon; (**d**) microstructure of the quinary alloy synthesized by powder metallurgy (P/M) [67], with copyright permission from © Elsevier S.A.

Synthesis of ferromagnetic Heusler alloys by P/M is often not the preferred choice of processing because it resulted in incomplete martensitic transformation [66] or evolved secondary phases [70], which are detrimental to the magnetostructural transformation and properties. Nevertheless, application of P/M in the case of ferromagnetic Heusler alloys opens opportunities for interested researchers. With pressureless sintering of porous samples of Ni_43_Co_7_Mn_39_Sn_11_ alloy powders at 1173 K, the porosity fraction decreased with the increase in the sintering time, from 65% at 12 h to 5% at 144 h [68]. Even though the microstructures in Figure 10a,b exhibited single-phase structures, still the shape memory property of the 12 h sintered sample was superior because dense specimens with grains surrounded by neighboring grains impose constraints during martensitic transformation. The microstructure of the same alloy composition sintered by spark plasma sintering is shown in Figure 10c. Concentrations of a precipitate phase rich in Co were observed. The compositional inhomogeneity due to the presence of a second phase and contamination by the graphite die during the spark process resulted in an imperfect shape memory effect [70].

It is well known that P/M methods are advantageous in terms of composition control. Furthermore, use of elemental powders instead of alloy powders can ensure substantial composition control while, at the same time, making P/M processes more cost-effective. Elemental powders, in addition, are easier to mix than alloy powders, which are harder and with poor compaction characteristics. Synthesis and characterization of a quinary Ni-Co-Mn-(Sn,Cu) alloy from elemental powders using P/M amply demonstrated the usefulness of the solid state processing method [67]. The alloy exhibited martensitic transformation, 6*M* martensite and magnetic field-induced transformation, seen in Figure 10d. However, analogous to the spark plasma sintered sample [70], the addition of a fifth element to the quaternary alloy, the existence of a multiphase microstructure (shown by red arrows) and grain growth affected the magnetostructural characteristics, which were reflected in the saturation magnetization not being similar to the bulk alloy. Thus, for solid state processing to become the preferred choice of synthesis of ferromagnetic shape memory alloys, compositional homogeneity through elimination of second phases by secondary heat treatment and inhibiting grain growth by secondary thermomechanical treatments are critical for identical magnetostructural characteristics, as in bulk alloys, to be apparent.

In the combinatorial approach of materials synthesis, a large number of different materials are synthesized by advanced fabrication methods on a single substrate under identical conditions and are subsequently screened for properties by parallel or fast sequential methods of high-throughput characterization. Combinatorial thin-film fabrication methods include physical vapor deposition, chemical vapor deposition, ion implantation and other continuous/discrete composition methods. Automated energy dispersive spectroscopy, X-ray diffraction, focused ion beam machining for TEM investigation and temperature-dependent magnetoelectronic property measurement are some of the high-throughput characterization techniques. Critical reviews of the combinatorial thin-film materials science [57] and combinatorial approaches for the high-throughput characterization of mechanical properties [112] add to the understanding.

**Figure 11:**
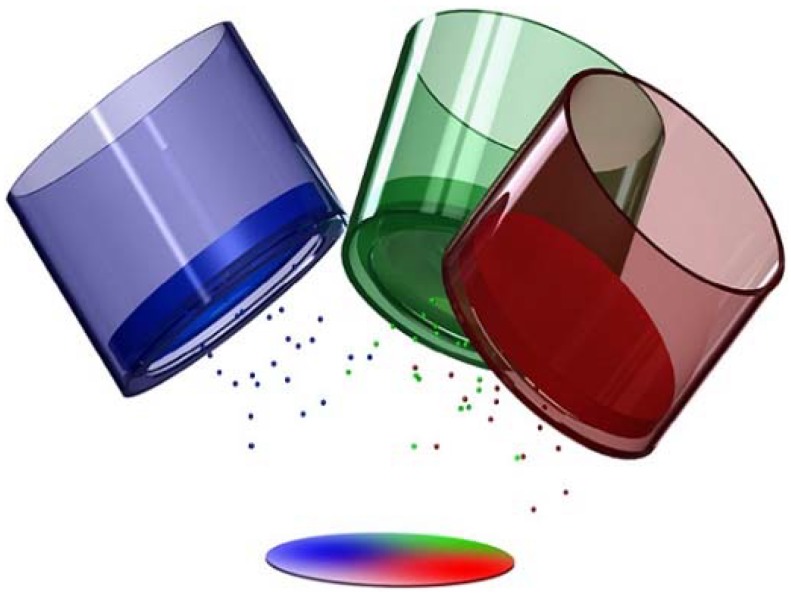
Schematic of the composition-spread method with three vapor sources [57], with copyright permission from © Elsevier S.A.

Composition-spread method, which is a continuous composition technique of thin-film synthesis based on co-deposition, is the most common synthesis method employed on shape memory and ferromagnetic shape memory materials. The schematic of the technique is shown in Figure 11.

This system is capable of sequential sputtering with six magnetron cathodes arranged along a movable arm sequentially depositing layered films on a substrate. Four computer-controlled shutters move during the deposition to create wedge-shaped thickness gradients across the substrate [113].

**Figure 12:**
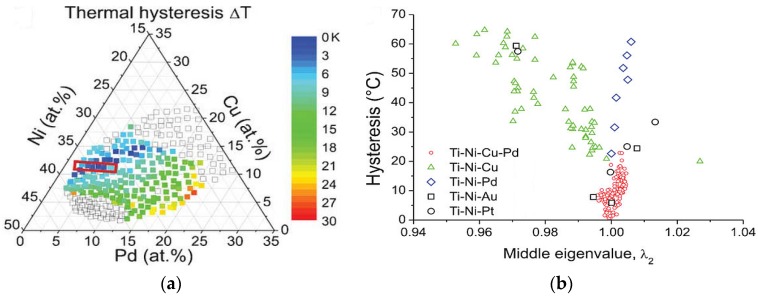
(**a**) ΔT values on a pseudoternary projection of the quaternary composition tetrahedron with the red box highlighting compositions for which ΔT approached zero; (**b**) thermal hysteresis values in Ti–Ni-based ternary and quaternary shape memory alloys [116], with copyright permission from © IOP Publishing.

Phase transformation characteristics of conventional shape memory alloys systems Ti-Ni-Cu and Ti-Ni-Pd for microactuator applications were investigated using composition-spread technique and high-throughput characterization by cantilever deflection methods and automated measurements [113,114]. With GNLTM, a combinatorial approach in ternary Ni-Ti-Cu and Ni-Ti-Pd systems [115], as well as in quaternary Ti-Ni-Cu-Pd shape memory alloy systems [116], yielded alloy compositions with thermal hysteresis width (ΔT = A_f_ − M_s_) converging to zero, close to $\lambda_{2}=1$. Figure 12a shows thermal hysteresis width values on a pseudoternary Ni-Cu-Pd phase diagram, which itself is a projection of the quaternary composition tetrahedron with Ti content limited to 47–67 atom %. Compositions with near-zero ΔT are shown in blue. Figure 12b shows ΔT vs. $\lambda_{2}$ plots of various ternary and quaternary alloy compositions, with the ΔT values converging to zero for quaternary compositions close to $\lambda_{2}=1$.

**Figure 13:**
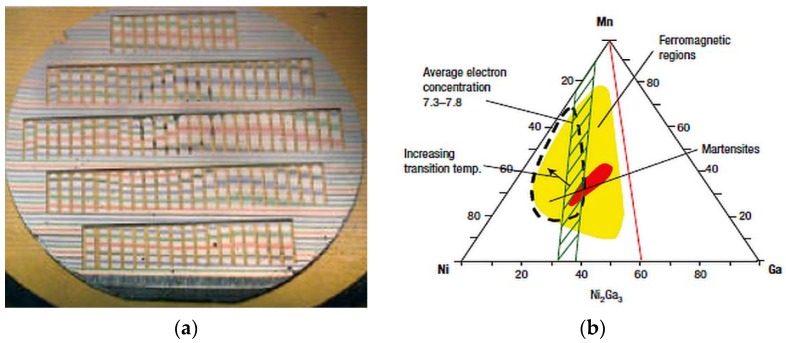
(**a**) Photograph of the cantilever library; (**b**) functional phase diagram of the Ni-Mn-Ni_2_Ga_3_ alloy system [117], with copyright permission from © Nature Publishing Group.

Thin-film composition-spreads deposited on micromachined arrays of cantilevers were screened using scanning superconducting quantum interference device (SQUID) microscope and X-ray microdiffractometer to map the functional phase diagram of the Ni-Mn-Ga ferromagnetic shape memory alloy system [117]. Figure 13a shows the Ni-Mn-Ni_2_Ga_3_ spread deposited on the cantilever library. Figure 13b shows the functional diagram, with the hatched region comprising compositions with average *e*/*a* ratio of 7.3–7.8, dotted line surrounding the region of reversible martensites and the red region having the highest magnetization in the yellow ferromagnetic region. A similar study was conducted on Ni-Mn-Al alloy system, where it was seen that both ferromagnetic and shape memory properties coexisted [118]. High-throughput screening using nanoindentation was performed on Ni-Mn-Al thin-film composition spreads to delineate martensitic regions, which were found to have low elastic modulus and hardness [119].

### 3.2. Characterization

In this section, we discuss how the different techniques of characterization have contributed to the understanding of several issues concerning magnetostructural behavior in ferromagnetic Heusler alloys from physical and metallurgical perspectives.

Differential scanning calorimetry (DSC) is a technique of thermal analysis which measures, depending on whether heat is absorbed or liberated, the enthalpy of phase transformation in a structurally transforming material as a function of time and temperature. It is often the first step in the characterization sequence, used for the determination of the martensitic and magnetic transition temperatures, heat flow curves, thermal hysteresis and enthalpy/entropy changes in ferromagnetic Heusler alloys [120,121,122,123]. Compositional dependence of martensitic transformation [124], progressive evolution of the martensitic transformation behavior in response to increasing Co content in Mn_50_Ni_40-x_In_10_Co_x_ [125] and the mechanism of suppression and recovery of martensitic transformation in NiCoMnIn alloys fabricated under nonequilibrium conditions [126] have been established using DSC. The suppression occurs when dendrite-like precipitates hinder the martensitic transformation [126].

**Figure 14:**
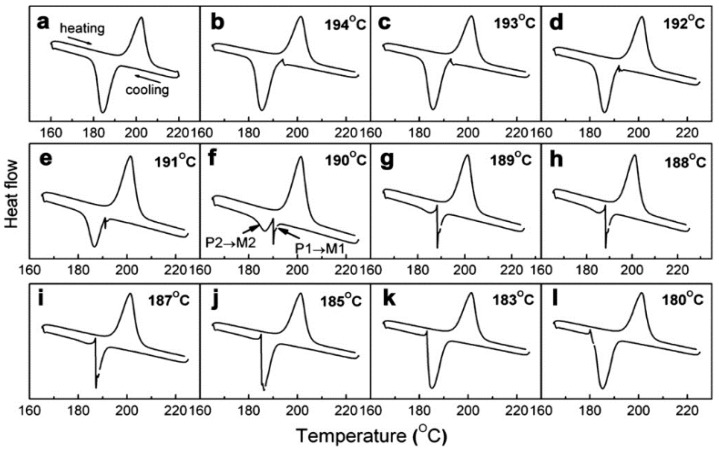
DSC curves of the transformation behavior of the Ni_43_Co_7_Mn_41_Sn_9_ alloy measured: (**a**) after a full transformation cycle (**b**–**l**) and during interrupted cooling [50], with copyright permission from © Elsevier Ltd.

An irreversible transformation of face-centered tetragonal (f.c.t) martensite to a body-centered martensite (b.c.t) at low temperature in a martensite-to-martensite transformation in Fe–Pd alloys has been captured using DSC [127]. Subsequent aging treatments stabilize the f.c.t martensite at low temperatures, which again has been determined using DSC [127]. The isothermal (time-dependent) nature of martensitic transformations was observed from elaborate interrupted forward and reverse transformation sequences carried out using DSC [50]. The transformation behavior of Ni_43_Co_7_Mn_41_Sn_9_ alloy after a full transformation cycle is shown in Figure 14a, and during interrupted cooling in Figure 14b–l. The interrupted transformation is shown as comprising two stages—part P1 (austenite) to M1 (martensite), and the remaining part P2 (austenite) to M2 (martensite) later during the interrupted process.

**Figure 15:**
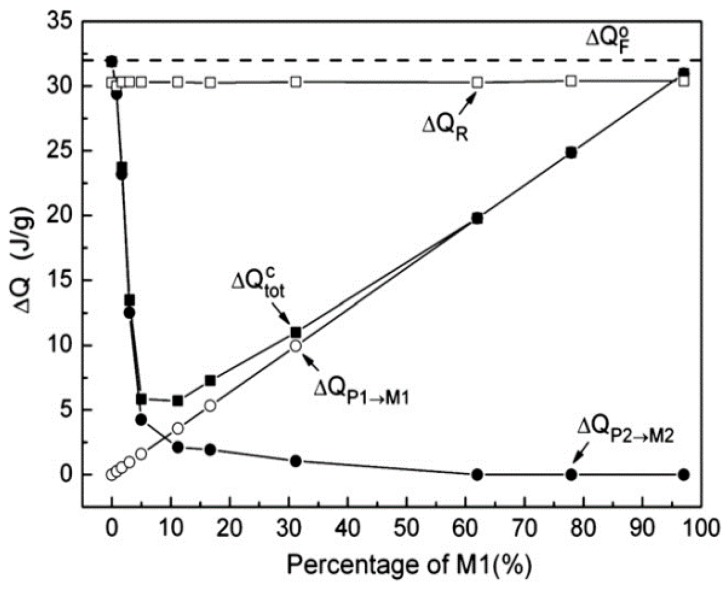
The absolute value of latent heat of P1–M1 and P2–M2, total latent heat and the reverse latent heat after interrupted cooling as a function of M1 percentage [50], with copyright permission from © Nature Publishing Group.

**Figure 16:**
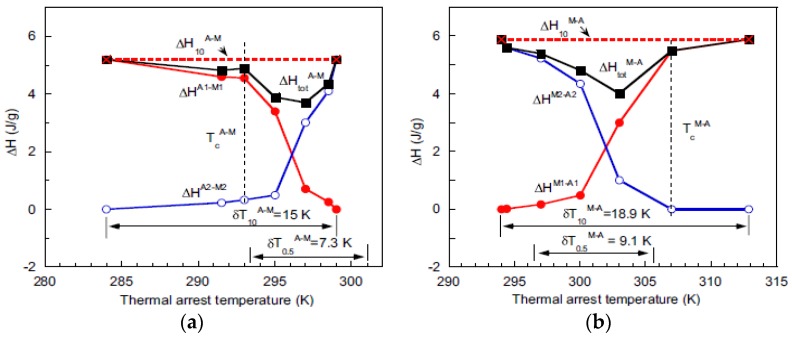
Absolute values of latent heat determined by interrupted measurement at different stages during: (**a**) forward and; (**b**) reverse L2_1_-orthorhombic martensitic transformation in Ni_43_Co_7_Mn_39_In_11_ at a cooling/heating rate of 10 K/min [128], with copyright permission from © Elsevier Ltd.

Figure 15 shows the latent heat values of P1–M1, P2–M2, total latent heat and reverse latent heat after interrupted cooling as a function of amount of M1 formed. Not shown is a similar interrupted heating process. From both the sequences it was seen that the transformations continued to completion (ΔQR constant in all interrupted cycles in Figure 15) demonstrating the time-dependence of austenite–martensite transformation at finite cooling rates [50]. However, the authors refute the findings that the time-dependent sequences of the transformations are just “artefacts” in which the latent heats of transformation dissipate by “inertia” continuation during thermal equilibration, which again are due to metallurgical causes. Identical experiments on NiTi and NiCoMnIn alloys [128] evaluate the latent heats, shown in Figure 16, of transformation in a supposedly thermal inertia-driven isothermal behavior.

Microscopy, including in situ microscopy and diffraction techniques, provides insight into the field-induced effects in both real and reciprocal space, respectively. Metallurgical samples are usually examined in reflection because the conduction electrons render metals opaque to visible light [129] and, consequently, the contrast seen in optical micrographs is topological. The characterization of martensitic structures as L1_0_, 14*M*, 10*M* and L2_1_ for x=0.05, 0.10, 0.13 and 0.25, respectively, in Ni_0.50_Mn_0.50−x_Sn_x_ alloys [130] and/or multiphase microstructures in Ni_45_Co_5_Mn_40_Sn_10_, Ni_44.5_Co_5.5_Mn_39.5_Sn_10.5_ and Ni_43_Co_7_Mn_39_Sn_11_ alloys, which included an fcc γ-phase representing decreases in Ni and Sn and increases in Mn and Co in both as-cast and annealed samples [73], are examples wherein optical microscopy has been put into good use.

**Figure 17:**
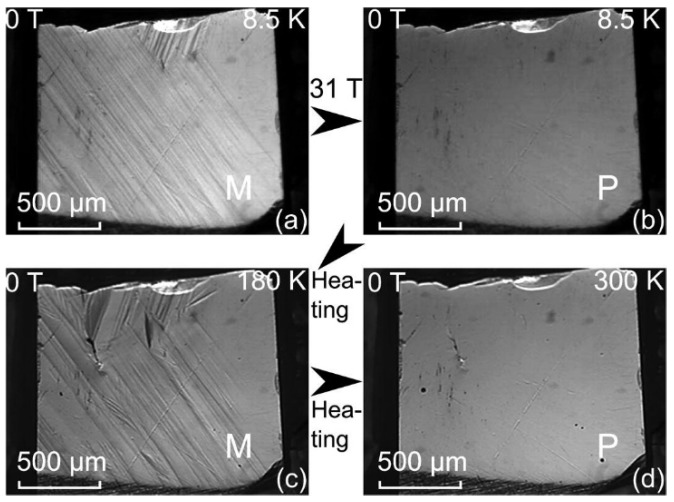
Micrographs: (**a**) before and (**b**) after a 31 T pulsed magnetic field application at 8.5 K. Parent phase (P—austenite) was arrested even when the magnetic field became zero in (**b**); (**c**) martensite phase obtained at 180 K out of an abnormal heating-induced transformation; (**d**) the martensite phase eventually transferred back to parent phase when further heated to 300 K [131], with copyright permission from © Pergamon.

In situ optical microscopy conducted on Ni_45_Co_5_Mn_36.7_In_13.3_ alloys studies the magnetic field-induced transformation (MFIT) and heating-induced martensitic transformation (HIMT), also known as kinetic arrest (KA) [131]. During the field-induced transformation from martensite to parent phase, the parent phase freezes upon removal of the applied field and the reappearance of martensite phase is not until the sample is heated. A high-speed microscopic imaging system (HSMIS) capable of in situ microscopic examination under a pulsed high magnetic field and at extremely low temperatures [132] has been used in this study. Figure 17 shows how the parent phase freezes when the magnetic field became zero and unfreezes upon heating to 180 K, giving rise to heating-induced martensitic transformation.

X-ray and neutron diffraction techniques measure in reciprocal space and provide an understanding of the typical states of identical entities, such as martensitic twin variants. The diffraction spectrum from a crystalline material during X-ray diffraction testing can be represented by the Bragg condition λ = 2dsinθ, where λ is the wavelength of the incident wave, d is the interplanar lattice spacing and θ is half the angle between the incident and the scattered beam. The distance *d* between planes is a function of the Miller indices of the planes and the lattice parameters of the crystal lattice [133]. With a polychromatic incident beam, individual Bragg reflections from lattice planes with same interplanar spacing of d are used to characterize similar microstructural entities and monitor twin reorientation [26]. To measure magnetic moments of the individual twin variants, neutron scattering is best suited, as the neutrons carry a spin [26] and with spin-polarized neutrons the rotation of magnetic moments can be separated from the crystallographic twin reorientation.

Small-angle neutron scattering has been used to observe the existence of nanosomic magnetic clusters at low temperatures in ferromagnetic shape memory alloys, which, along with the magnetometry data, helps in understanding physical phenomena such as nanoscale magnetic inhomogeneity, spatial distribution of clusters, their mean spacing and diameter, nature of magnetic order in martensite matrix and the spatial extent of the intercluster magnetic interactions [134]. The techniques of characterization complement each other, as can be seen from the results of neutron diffraction experiments which confirm the martensite-to-martensite transformation in Fe–Pd alloys identified using DSC [127]. High-resolution neutron diffraction experiments are usually performed to study changes in crystallographic structure with respect to temperature. An example of this kind of study is found in [135], where over a temperature range from 400 K to 20 K, the lattice parameters for the transformation sequence of austenite (L2_1_)–pre-martensite (3O)–martensite (7O) were established.

**Figure 18:**
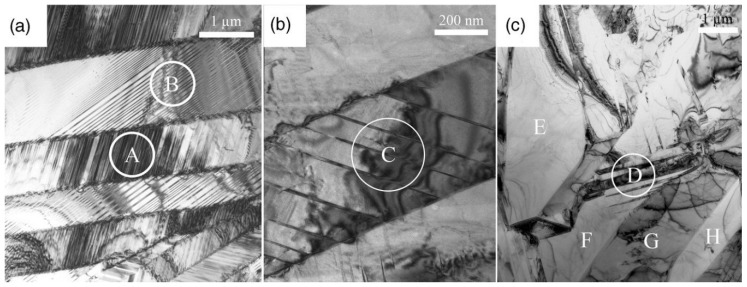
Evolution of microstructure with composition: (**a**) internally twinned martensite plates in Ti_50_Ni_25_Pd_25_, A, B; (**b**) plate in Ti_50_Ni_30_Pd_20_ with a smaller twin ratio, C; (**c**) example of microstructure in Ti_50_Ni_39_Pd_11_ composed of a mosaic of twinless martensite plates (denoted E, F, G, H) and a group of compound twins (D) [138], with copyright permission from © Taylor and Francis.

Scanning electron microscope has a depth of field for resolved detail much greater than the spatial resolution in the field of view and, consequently, the flatness of the topological and morphological detail in the optical or transmission electron microscope (TEM) is replaced by an image that is very similar to the play of light and shade over hills and valleys [136]. TEM, on the other hand, extends the resolution available for morphological studies to the order of 0.1 nm or even sub-angstrom. TEM combines real space data at excellent resolution and the information from reciprocal space, i.e., electron diffraction patterns, can be recorded [137]. TEM has been used to investigate the evolution of martensite morphology, shown in Figure 18, as the composition of Ti_50_Ni_50−x_Pd_x_ system is tuned to achieve geometric compatibility at the austenite–martensite interface [55,138]. The perfect one-to-one correspondence between the antiphase boundaries (APBs) and the magnetic domain walls in the parent phase of Ni2Mn(Al, Ga) has been studied by combining Lorentz TEM [139].

**Figure 19:**
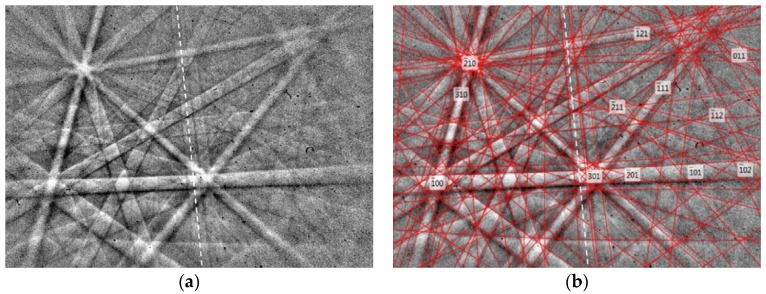
(**a**) Measured and (**b**) simulated electron backscattered diffraction (EBSD) Kikuchi patterns. One of the satellite reflections is highlighted with a white dashed line [142], with copyright permission from © Elsevier.

The use of electron backscattered diffraction (EBSD) to determine the orientation information of the martensitic lamellae is demonstrated in [140,141]. EBSD patterns of bulk Ni_2_Mn_1.44_In_0.56_ are shown in Figure 19a for an incommensurate 6*M* modulated martensite, and the calculated Kikuchi lines (solid red) can be seen from Figure 19b [142]. The main and satellite reflections are highlighted by the white dashed line.

Magnetization measurements are usually carried out by magnetometry on a superconducting quantum interference device (SQUID) magnetometer or a vibrating sample magnetometer (VSM) to establish the temperature dependence of magnetization M(T) in applied magnetic fields. The SQUID magnetometer can detect incredibly small magnetic fields of the order of fields in living organisms [143]. Most of the SQUID magnetometers have a temperature range of 0 K≤T≤400 K, while measurements up to 700 K are also possible. The measurements are done in sequential zero-field-cooled (ZFC), field-cooled (FC) and field-heated (FH) protocols. During ZFC, the specimen is cooled from higher temperature to lower temperature without the application of the magnetic field. The specimen is then heated to higher temperature in the presence of a magnetic field while recording the magnetization values with increasing temperature. With the magnetic field still present, the specimen is cooled to lower temperature for the FC protocol. The specimen is subsequently heated in the applied magnetic field to a higher temperature for an FH protocol.

**Figure 20:**
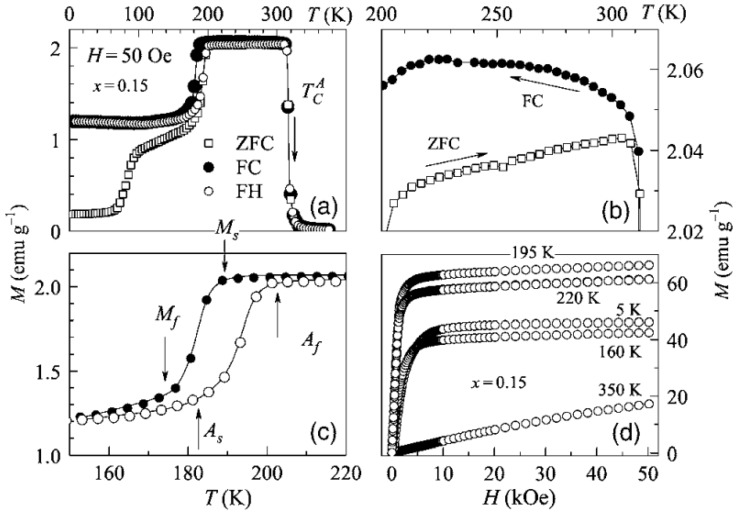
(**a**–**d**) Temperature-dependent magnetization (M(T)) and magnetic-field-dependent magnetization (M(H)) curves of Ni_0.50_Mn_0.35_Sn_0.15_ alloy [130], with copyright permission from © IOP Publishing.

An observed hysteresis in the FC and FH curves is indicative of structural transition, while the observed split between the ZFC and FH curves is surmised as the effect of pinning by antiferromagnetic (AF) or non-collinear magnetic structures present in the ferromagnetic (FM) matrix or in the twin boundaries of martensite. The magnetic properties are further characterized by measuring the magnetic field dependence of magnetization M(H) in applied fields up to 50 kOe. Examples of magnetic characterization of Ni_0.50_Mn_0.50−x_Sn_x_ for *x* = 0.15 by SQUID magnetometry are shown in Figure 20a–d [130].

From Figure 20a, it can be seen the sample is ferromagnetic (FM) below $T_{C}^{A}=320\;K$ down to about 190 K, beyond which the magnetization decreases. Figure 20b shows the splitting between the ZFC and FC, which is wider in the martensitic state because the lattice distortions in the martensitic state greatly influence the pinning of non-collinear magnetic configurations in the ferromagnetic domain. Hysteresis observed near 190 K, as seen in Figure 20c, indicates a first-order structural transition, and M(H) curves in Figure 20d indicate the samples are ferromagnetic below the martensitic temperature.

## 4. Microstructural Effects on Properties

In this section, the various microstructural factors which affect all or any of the magnetostructural properties of ferromagnetic Heusler alloys are discussed. It begins with a description of the effect of composition on the properties.

The most important aspect concerning the magnetostructural effects, microstructures, properties and functions of ferromagnetic Heusler alloys is the composition. Ni_54_Mn_20_Ga_26_ thin films prepared by magnetron sputtering demonstrated martensitic phase transformation above room temperature [144] because of a greater extent of hybridization between the excess Ni and Ga in the alloy besides film stress. The effect of composition is not restricted to the prototype Ni-Mn-Ga alloys alone. Metamagnetic shape memory alloys are growing to be technologically significant [100,124,145,146,147,148], where the stoichiometric composition is varied by tuning the compositions at will with remarkable magnetocaloric effects. To cap it all is the tuning of the composition to draw a perfect austenite–martensite interface with no stressed transition layer, which yielded the highest saturation magnetization several orders higher than normally observed [54]. The alloy Ni_45_Co_5_Mn_40_Sn_10_ resulting from the tuning was later used in direct energy conversion [25].

While the crystal structure has a certain effect on the magnetostructural properties, tuning the composition changes it, affecting the property being studied. The exact martensitic structure that would evolve for a particular composition and its effect on the properties could not be predicted, as there are no rules to predict them. Nevertheless, the structure may evolve as cubic–10*M*–14*M*–L1_0_, as seen earlier, in accordance with an increasing *e*/*a* ratio and temperature [2].

In the case of Ga-doped NiMn alloys, for the structure to be technologically significant, the twinning stress has to be minimum, for which the lattice distortion, *c*/*a*, of the martensite has to be minimum. 5*M* and 7*M* martensites were suitable for MFIS by virtue of the low twinning stress and high anisotropy in them, while the nonlayered tetragonal *T*(L1_0_) reportedly exhibited high anisotropy and high twinning stress, but not MFIS [149]. However, addition of small amounts of Co and Cu to NiMnGa [150], as shown in Table 1, yielded a non-modulated structure with an MFIS of 12%.

Table 2 shows a list of example MC alloys along with the magnetic entropy change ΔS_m_ values.

The differences in ΔS_m_ in off-stoichiometric Ni-Co-Mn-Sn alloys are due changes in composition, as seen from arc-melted Ni_41_Co_9_Mn_40_Sn_10_, Ni_43_Co_7_Mn_39_Sn_11_ and Ni_50_Mn_34_Co_2_Sn_14_ alloys and melt-spun Ni_48_Co_2_Mn_38_Sn_12_ alloy. The composition apparently overrides the crystal structure in the determination of the properties. It may be seen that intermartensitic or martensite-to-martensite transformations yield intermediate structures which may undergo irreversible transformation to b.c.t structures capable of deteriorating the MSM effect [127]. On the other hand, steplike thermoelastic martensite transformations are likely to enhance magnetocaloric properties [64]. Intermartensitic and steplike transformations, however, are still not clearly understood [149].

**Figure 21:**
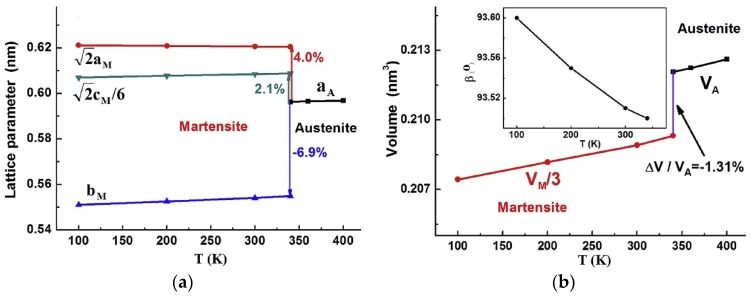
(**a**) Effect of lattice parameters; (**b**) unit cell volume change with respect to temperature [146], with copyright permission from © Elsevier BV.

Across the martensitic transformation, a change in the unit cell volume has a profound effect on the magnetostructural properties, particularly magnetocaloric, and also on the martensitic transformation [160,161,162]. One has to know the orientation relationship between austenite and martensite to better understand the effect of lattice and, consequently, the volume change, as can be seen from Figure 21, where the variations of $\sqrt{2}a_{M}$, $\sqrt{2}c_{M}/6$ and $b_{M}$ (instead of $a_{M}$, $c_{M}$ and $b_{M}$) and $V_{M}/3$ (instead of $V_{M}$) are plotted. The lattice can be seen to expand along $a_{M}$ and $c_{M}$ axes and shrink along $b_{M}$ axis. The volume contraction is −1.31%, accounting for a magnetic entropy of 31.9 J/kgK [146]. Similarly, a volume change of 4% accounted for an entropy change of −47.3 J/kgK in Mn-Co-Ge-B alloy [163,164]. This correlation between the unit cell volume change, structural entropy change and magnetic entropy change [163] can be used to predict high-performance magnetocaloric materials from crystallographic data [146].

The degree of long-range L2_1_ atomic order significantly lowers the transformation entropy change through large shifts in the transformation temperatures in relation to the change in magnetization of the austenite phase [165,166,167,168]. While L2_1_ atomic order refers to the fraction of atoms located in the correct sublattice of L2_1_ structure, studies show it can be varied by thermal treatment. Thermal treatments are better than post-quench, as quenching does not affect the retained degree of L2_1_ atomic order to be able to have appreciable effect on the martensitic transformation [169]. Post-quench aging (post-quench atomic ordering), as in polycrystalline Ni_45_Mn_36.7_In_13.3_Co_5_, has the effect of increasing the difference between the austenite Curie temperature (due to improved ferromagnetic coupling between Mn atoms) and the martensitic transformation temperature, thereby lowering the martensitic entropy change [169].

**Figure 22:**
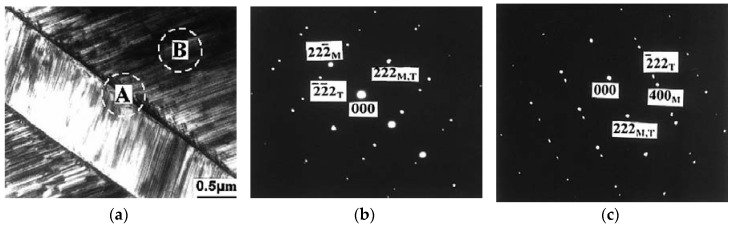
(**a**) TEM bright-field image of Ni_54_Mn_25_Ga_21_ alloy (**b**); SAED pattern in the [1$\bar{1}$0] taken from area A in (**a**); (**c**) SAED taken from area B in (**a**) with the incident electron beam tilted to [0$\bar{1}$1] [171], with copyright permission from © Elsevier.

Microstructural changes through tuning of stoichiometry also have the effect of increasing the transformation temperatures, thus transporting the alloys into the realm of high-temperature shape memory alloys (HTSMAs, with temperatures up to 300 °C above the standard SMAs, such as TiNi with operating temperature of 100 °C). The structure remains cubic L2_1_ at higher temperatures and transforms to tetragonal upon cooling. Such alloys are useful for applications in automotive and aerospace engines, limited by issues such as brittleness of the alloys. The formation of a ductile γ phase with [84,170] or without [171] addition of dopants like Co improves the ductility of the alloy, however, it does not contribute to shape memory effect (SME). The loss of SM/MSM is offset by an improvement in the mechanical properties [171]. No SME is observed when the martensite is two-phase, including γ phase. On the contrary, the single-phase martensite with no γ phase in Ni_54_Mn_25_Ga_21_ recorded a good SME, whose microstructure shown in Figure 22a consists of coarse (1–5 µm) and fine (0.05–0.1 µm) microtwins, both twinned along [111] directions, which strongly favors thermoelasticity.

Where there has been an appreciable shape memory effect (SME) in two-phase martensite with γ phase, it is because the grain size of the γ phase is smaller, with circular morphology brought about by annealing [170] and thermomechanical [172] treatments. The stress–strain behavior in these alloys is characterized by dislocation slip and high strain hardening.

**Figure 23:**
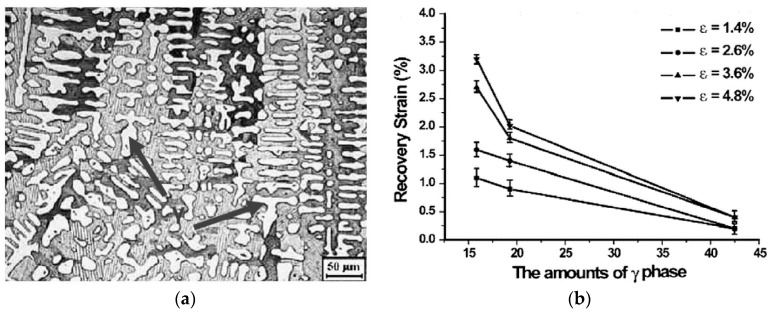
(**a**) Optical micrograph of Ni_56_Mn_17_Co_8_Ga_19_ with highest γ phase content; (**b**) shape memory effect in relation to the volume content of γ phase [84], with copyright permission from © Elsevier.

It is important also to note that γ phase is dependent on whether the Co atoms substitute Ni alone or Mn alone or both Ni and Mn. Figure 23a shows the microstructure of Ni_56_Mn_17_Co_8_Ga_19_ alloy (Co substituted for Mn) with the highest volume of γ phase amongst the various alloys investigated, wherein the γ phases join together to form slender bars (black arrows), and Figure 23b shows the recovery strain decreasing to almost zero at the highest γ phase content of 43% for various values of prestrain [84]. This was because the γ phase affected reversible martensitic transformation by hampering the martensite reorientation. To retain the MSME at high temperatures even though the temperatures are not as high as conventional SME, the choice of the doping element has to be judicious.

While rare earth elements like Gd, Tb or Nd serve the purpose [173] in addition to improving the mechanical properties by way of grain refinement, the most suitable high-temperature magnetic shape memory alloy so far has been reported as the six-element Ni_45_Co_5_Mn_21_Fe_4_Ga_20_Cu_5_ alloy [174], in which doping with Co, Cu and Fe instead of Co/Cu or Fe/Co increased the martensitic transformation temperature to 400 K and a Curie temperature to 458 K.

The microstructures of the alloys which wield enormous influence on their magnetostructural properties are greatly dependent on the heat treatment methods adopted. Long-period annealing achieves structural and chemical homogenization [21], after which the magnetostructural behavior is conspicuous. The reasons are understandable because multiphase solidification leads to compositional variations which persist even after short anneals and are capable of annulling the magnetostructural behavior [76]. Annealing of Ni_48_Mn_39_In_13_ ribbons caused changes in the atomic ordering and the grain size of the alloy ribbon, due to which the transition temperatures and magnetic entropy change increased considerably [175]. Low-temperature annealing of homogenized samples (1223 K for 4 weeks) of Ni_50_Mn_50−x_Sn_x_ (*x* = 10–25) alloys at 773 K caused decomposition to compositions near *x* = 1 and *x* = 20. While one composition would likely exhibit transformation above 700 K, the other exhibited none [74,176]. It was confirmed that the L2_1_ phase is metastable at 773 K over an intermediate composition range and that transformations below 400 K account for the ferromagnetic shape memory behavior.

The combination of processes and heat treatments also contribute to the microstructure. Heat-treated ribbons exhibited ~1.7 times larger GS/*t* (grain size to thickness ratio) than solutionized bulk sample and were more suited for magnetocaloric applications [96]. Wheel speed and annealing temperature have been reported to be controlling factors for grain size, interatomic distance, smaller thermal hysteresis and degree of atomic ordering in order to achieve remarkable magnetic properties, such as high values of magnetic entropy change (ΔS_M_) and refrigeration capacity (RC) [94,177]. There are, however, conflicting claims that in alloys in which L2_1_–B2 ordering transition is absent, thermal treatments are in vain with regards to the tuning of the magnetostructural properties [178], while high-temperature annealing has been reported to favor the tuning of the degree of atomic order in Ni_49_Mn_39_Sn_12_ alloy ribbons with L2_1_–B2 ordering [94]. This aspect requires further investigation.

In the case of microwires, the inhibiting influence of internal stresses on the magnetostructural characteristics are compensated by annealing them, with and without the glass coating. Internal stresses are induced during solidification and by the difference in the coefficients of thermal expansion of the glass coating and the metallic alloy. Microwires annealed after glass coating removal showed an increased value of saturation magnetization (495 emu/cm^3^) [89]. However, their remanence (245 emu/cm^3^) and coercivity (248 Oe) are decreased in comparison with the microwire annealed with glass coating first and then after removal. Relaxation of the induced stresses in the microwire improved the atomic ordering, thus reducing the coercivity and remanence. On the other hand, high-temperature annealing of Ni_63_Mn_12_Ga_25_ microwire resulted in a partial evaporation of Mn from the outer region of the metallic core [90]. This combined with the glass melting led to the formation of MnO_x_ at the surface. The middle part consisted of Mn-depleted Ni_3_Ga and an L2_1_ structure with a composition of Ni_60_Mn_9_Ga_31_. Such a structure resulted in a soft ferromagnetic behavior below 270 K [90].

## 5. Summary

In this paper, we have reviewed the Ni–Mn-based ferromagnetic Heusler alloys from the perspective of design, synthesis, characterization and structure–property relationships.

- On the basis of design, given that NiMn-based Heusler alloys are multiferroic and multifunctional, adaptation of cofactor conditions from GNLTM will certainly be useful in arriving at highly superior multifunctional compositions which can be readily used in applications. Combinatorial approach combined with GNLTM has the potential to revolutionize materials search by accelerating discovery and optimization of new and known materials.
- Processing methods and techniques have an influence on the MSM/MMSM and MC properties of these materials through benign changes in their microstructures. Every method has its advantages and disadvantages. While liquid processing ensures homogeneity of the alloys, issues such as multiphase solidification and chemical segregation, which tend to cover up the magnetostructural transitions, still persist. Melt-spinning can be useful in terms of homogeneity of grain size and avoidance of annealing but may still require secondary treatment for the realization of desired characteristics. In view of this, exclusion of a particular method, like P/M, may not be justified. The disadvantages of incomplete martensitic transformation, formation of secondary and intermetallic phases, etc., which are characteristic of P/M, can be overcome by appropriate heat treatment or thermomechanical procedures and through exact characterization of the starting powders.
- Various factors, such as composition, crystal structure, atomic ordering, volume of unit cell, grain size, presence or absence of secondary phases and heat treatment methods, which influence the MSM/MMSM and MC effects of these alloys, have been reviewed. The overriding factor is the composition, which influences both martensitic and magnetic transformations, the transformation temperatures, crystal structures, saturation magnetization and consequently the magnetostructural effects.

## Figures and Tables

**Table 1:** Crystal structure and magnetic field-induced strain (MFIS) in magnetic/metamagnetic shape memory (MSM/MMSM) alloys retrieved from literature.

Example Alloys	Process	Crystal Structure	MFIS	Ref.
Ni_49.8_Mn_28.5_Ga_21.7_	Bridgman	bct, *I*4/*mmm*	6%	[6]
Ni_48.8_Mn_29.7_Ga_21.5_	Bridgman	7*M* orthorhombic	9.50%	[5]
Ni_2_MnGa	Single crystal	bct	0.20%	[3]
Ni_506_Mn_28.3_Ga_21.1_	Arc-melting	7*M*	500 ppm	[151]
Ni_48.0_Mn_30.6_Ga_21.5_		5*M*	160 ppm	
Ni_46_Mn_24_Ga_22_Co_4_Cu_4_	Induction melting	NM	12%	[150]
Ni_45_Co_5_Mn_36.7_In_13.3_	Induction melting	L2_1_ and 14*M*	3%	[11]
Ni_43_Co_7_Mn_39_Sn_11_	Induction melting	L2_1_ and 10*M*/6*M*	1.00%	[12]
Ni_45.7_Co_4.8_Mn_35.6_In_13.8_	Bridgman	L2_1_	3.10%	[152]
Ni_45_Mn_36.5_Co_5_In_13.5_	Induction melting	L2_1_ and 6*M*	1.20%	[153]
Ni_0.50_Mn_0.34_In_0.16_	Arc-melting	10*M*	0.12%	[123]
Ni_46_Co_4_Mn_39_Sn_11_	Arc-melting	L2_1_	0.01%	[154]
Ni_50_Mn_34_In_16_	Density functional theory using PBE formulation	-	−4.5%	[155]
Ni_44.9_Co_5.1_Mn_37.5_In_12.5_		-	−5.1%	
Ni_40.9_Co_9.1_Mn_37.5_Sn_12.5_		-	−2.6%	
Ni_35.2_Co_14.8_Mn_37.5_Sb_12.5_		-	−2.7%	

**Table 2:** Crystal structure and ΔS_m_ values in magnetocaloric (MC) alloys retrieved from literature.

Example Alloys	Process	Crystal Structure	ΔS_m_ (J/kgK)	Ref.
Ni_45_Mn_43_CrSn_11_	Arc-melting, annealed	Cubic and tetragonal	39.7	[24]
Ni_41_Co_9_Mn_40_Sn_10_	Arc-melting, annealed	6*M*	31.9	[146]
Ni_43_Co_7_Mn_39_Sn_11_	As-spun annealed	L2_1_ and L1_0_ L2_1_ and L1_0_	9.5 23.9	[99]
Ni_42.6_Mn_39.6_Sn_9.7_Fe_8.1_	Melt-spun	7M	11.0	[156]
Ni_42_Co_8_Mn_39_Sn_11_	Arc-melting, annealed at 1170 K, 14 d	L2_1_	-	[145]
Ni_49_Mn_39_Sn_12_	Melt-spun	L2_1_	8.2	[94]
Ni_50_Mn_34_Co_2_Sn_14_	Arc-melting	L2_1_	48.8	[157]
Ni_48_Co_2_Mn_38_Sn_12_	Melt-spun	Austenite	32	[100]
Ni_0.50_Mn_0.34_In_0.16_	Arc-melting	10*M*	12	[123]
Ni_50_Mn_33.66_Cr_0.34_In_16_	Arc-melting	Austenite and orthorhombic	17.7	[23]
Ni_49.5_Mn_25.4_Ga_25.1_	Single crystal	Austenite	~11	[17]
Ni_2.19_Mn_0.81_Ga	Arc-melting	Monophase	20	[16]
Ni_54.8_Mn_20.3_Ga_24.9_	Arc-melting	Non-modulated	−7.0	[158]
Ni_55_Mn_18.9_Ga_26.1_	Arc-melting	7*M*	−5.2	[158]
Ni_55.3_Mn_18.1_Ga_26.6_	Arc-melting	Austenite	−1.3	[158]
Ni_55_Mn_20.6_Ga_24.4_	Melt-spun	7*M*	−9.5	[159]
Ni_55_Mn_19.6_Ga_25.4_	Melt-spun	7*M*	−10.4	[159]

## References

[1] James R.D., Zhang Z., Manosa L., Planes A., Saxena A. (2005). A way to search for multiferroic materials with “unlikely” combinations of physical properties. Magnetism and Structure in Functional Materials.

[2] Planes A., Mañosa L., Acet M. (2009). Magnetocaloric effect and its relation to shape-memory properties in ferromagnetic Heusler alloys. J. Phys. Condens. Matter.

[3] Ullakko K., Huang J.K., Kantner C., O’Handley R.C., Kokorin V.V. (1996). Large magnetic-field-induced strains in Ni2MnGa single crystals. Appl. Phys. Lett..

[4] James R.D., Tickle R., Wuttig M. (1999). Large field-induced strains in ferromagnetic shape memory materials. Mater. Sci. Eng. A.

[5] Sozinov A., Likachev A.A., Lanska N., Ulakko K. (2001). Giant magnetic-field-induced strain in NiMnGa seven-layered martensitic phase. Appl. Phys. Lett..

[6] Murray S.J., Marioni M., Allen S.M., O’Handley R.C., Lograsso T.A. (2000). 6% magnetic-field-induced strain by twin-boundary motion in ferromagnetic Ni-Mn-Ga. Appl. Phys. Lett..

[7] Murray S.J. (2000). Large field induced strain in single crystalline Ni-Mn-Ga ferromagnetic shape memory alloy. J. Appl. Phys..

[8] Sozinov A., Likhachev A.A., Lanska N., Ullakko K., Lindroos V.K. (2002). Crystal Structure, Magnetic Anisotropy, and Mechanical Properties of Seven-Layered Martensite in Ni-Mn-Ga.

[9] Sutou Y., Imano Y., Koeda N., Omori T., Kainuma R., Ishida K., Oikawa K. (2004). Magnetic and martensitic transformations of NiMnX(X = In, Sn, Sb) ferromagnetic shape memory alloys. Appl. Phys. Lett..

[10] Krenke T., Duman E., Acet M., Wassermann E.F., Moya X., Manosa L., Planes A. (2005). Inverse magnetocaloric effect in ferromagnetic Ni-Mn-Sn alloys. Nat. Mater..

[11] Kainuma R., Imano Y., Ito W., Sutou Y., Morito H., Okamoto S., Kitakami O., Oikawa K., Fujita A., Kanomata T. (2006). Magnetic-field-induced shape recovery by reverse phase transformation. Nature.

[12] Kainuma R., Imano Y., Ito W., Morito H., Sutou Y., Oikawa K., Fujita A., Ishida K., Okamoto S., Kitakami O. (2006). Metamagnetic shape memory effect in a Heusler-type Ni_43_Co_7_Mn_39_Sn_11_ polycrystalline alloy. Appl. Phys. Lett..

[13] Planes A., Manosa L., Acet M. (2013). Recent Progress and future perspectives in magnetic and metamagnetic shape-memory Heusler alloys. Mater. Sci. Forum.

[14] Pecharsky V.K., Gschneidner K.A. (1997). Giant magnetocaloric effect in Gd_5_(Si_2_Ge_2_). Phys. Rev. Lett..

[15] Hu F.-X., Shen B.-G., Sun J.-R. (2000). Magnetic entropy change in Ni_51.5_Mn_22.7_Ga_25.8_ alloy. Appl. Phys. Lett..

[16] Pareti L., Solzi M., Albertini F., Paoluzi A. (2003). Giant entropy change at the co-occurrence of structural and magnetic transitions in the Ni_2.19_Mn_0.81_Ga Heusler alloy. Eur. Phys. J. B.

[17] Marcos J., Planes A., Manosa L., Casanova F., Batlle X., Labarta A., Martinez B. (2002). Magnetic field induced entropy change and magnetoelasticity in Ni-Mn-Ga alloys. Phys. Rev. B.

[18] Marcos J., Planes A., Manosa L., Casanova F., Batlle X., Labarta A. (2003). Multiscale origin of the magnetocaloric effect in Ni-Mn-Ga shape-memory alloys. Phys. Rev. B.

[19] Roy S.B. (2013). First order magneto-structural phase transition and associated multi-functional properties in magnetic solids. J. Phys. Condens. Matter.

[20] Franco V., Bl’azquez J.S., Ingale B., Conde A. (2012). The magnetocaloric effect and magnetic refrigeration near room temperature: Materials and models. Annu. Rev. Mater. Res..

[21] Schlagel D.L., Yuhasz W.M., Dennis K.W., McCallum R.W., Lograsso T.A. (2008). Temperature dependence of the field-induced phase transformation in Ni_50_Mn_37_Sn_13_. Scr. Mater..

[22] Moya X., Manosa L., Planes A., Krenke T., Duman E., Wassermann E.F. (2007). Calorimetric study of the inverse magnetocaloric effect in ferromagnetic Ni-Mn-Sn. J. Magn. Magn. Mater..

[23] Sharma V.K., Chattopadhyay M.K., Roy S.B. (2010). Large magnetocaloric effect in Ni_50_Mn_33.66_Cr_0.34_In_16_ alloy. J. Phys. D Appl. Phys..

[24] Pandey S., Quetz A., Aryal A., Dubenko I., Mazumdar D., Stadler S., Ali N. (2017). Large Inverse Magnetocaloric Effects and Giant Magnetoresistance in Ni-Mn-Cr-Sn Heusler Alloys. Magnetochemistry.

[25] Srivastava V., Song Y., Bhatti K., James R.D. (2011). The direct conversion of heat to electricity using multiferroic alloys. Adv. Energy Mater..

[26] Pramanick A., Wang X.-L. (2012). Characterization of magnetoelastic coupling in ferromagnetic shape memory alloys using neutron diffraction. JOM.

[27] Franco V., Blázquez J.S., Ipus J.J., Law J.Y., Moreno-Ramírez L.M., Conde A. (2018). Magnetocaloric effect: From materials research to refrigeration devices. Prog. Mater. Sci..

[28] Khovaylo V.V., Rodionova V.V., Shevyrtalov S.N., Novosad V. (2014). Magnetocaloric effect in “reduced” dimensions: Thin films, ribbons, and microwires of Heusler alloys and related compounds. Phys. Status Solidi B.

[29] Graf T., Felser C., Felser C., Fecher G.H. (2013). Heusler compounds at a glance. Spintronics From Materials to Devices.

[30] Pons J., Chernenko V.A., Santamarta R., Cesari E. (2000). Crystal structure of martensitic phases in Ni-Mn-Ga shape memory alloys. Acta Mater..

[31] Halder M., Mukadam M.D., Suresh K.G., Yusuf S.M. (2015). Electronic, structural, and magnetic properties of the quaternary Heusler alloy NiCoMnZ (Z = Al, Ge, and Sn). J. Magn. Magn. Mater..

[32] Srivastava V., Bhatti K.P., Virk H.S., Kleeman W. (2012). Ferromagnetic shape memory Heusler alloys. Ferroics and Multiferroics.

[33] Ito W., Imano Y., Kainuma R., Sutou Y., Oikawa K., Ishida K. (2007). Martensitic and Magnetic Transformation Behaviors in Heusler-Type NiMnIn and NiCoMnIn Metamagnetic Shape Memory Alloys.

[34] Khovaylo V.V., Kanomata T., Tanaka T., Nakashima M., Amako Y., Kainuma R., Umetsu R.Y., Morito H., Miki H. (2009). Magnetic properties of Ni_50_Mn_34.8_In_15.2_ probed by Mössbauer spectroscopy. Phys. Rev. B.

[35] Righi L., Albertini F., Fabbrici S., Paoluzi A., Chernenko V.A. (2011). Crystal structures of modulated martensitic phases of FSM Heusler alloys. Advances in Magnetic Shape Memory Materials.

[36] Bersuker I.B., Köppel H., Yarkony D.R., Barentzen H. (2009). Recent Developments in the Jahn–Teller Effect. The Jahn-Teller Effect. Fundamentals and Implications. For Physics and Chemistry.

[37] Haritha L., Gangadhar Reddy G., Ramakanth A., Ghatak S.K., Nolting W. (2010). Interplay of magnetic order and Jahn–Teller distortion in a model with strongly correlated electrons ystem. Physica B.

[38] Cong D.Y., Roth S., Schultz L. (2012). Magnetic properties and structural transformations in Ni-Co-Mn-Sn multifunctional alloys. Acta Mater..

[39] Sharma V.K., Chattopadhyay M.K., Roy S.B. (2007). Kinetic arrest of the first order austenite to martensite phase transition in Ni_50_Mn_34_In_16_: Dc magnetization studies. Phys. Rev. B.

[40] Zubar T.I., Panina L.V., Kovaleva N.N., Sharko S.A., Tishkevich D.I., Vinnik D.A., Gudkova S.A., Trukhanova E.L., Trofimov E.A., Chizhik S.A. (2018). Anomalies in growth of electrodeposited Ni–Fe nanogranular films. Cryst. Eng. Comm..

[41] Zubar T.I., Sharko S.A., Tishkevich D.I., Kovaleva N.N., Vinnik D.A., Gudkova S.A., Trukhanova E.L., Trofimov E.A., Chizhik S.A., Panina L.V. (2018). Anomalies in Ni-Fe nanogranular films growth. J. Alloy. Compd..

[42] Wang X., Shang J.-X., Wang F.-H., Jiang C.-B., Xu H.-B. (2014). Origin of unusual properties in the ferromagnetic Heusler alloy Ni–Mn–Sn: A first-principles investigation. Scr. Mater..

[43] Pérez-Reche F.J., Vives E., Mañosa L., Planes A. (2001). Athermal Character of Structural Phase Transitions. Phys. Rev. Lett..

[44] Zheng H., Wang W., Wu D., Xue S., Zhai Q., Frenzel J., Luo Z. (2013). Athermal nature of the martensitic transformation in Heusler alloy Ni-Mn-Sn. Intermetallics.

[45] Kakeshita T., Kuroiwa K., Shimizu K., Ikeda T., Yamagishi A., Date M. (1993). A New Model Explainable for Both the Athermal and Isothermal Natures of Martensitic Transformations in Fe-Ni-Mn Alloys. Mater. Trans. JIM.

[46] Faran E., Shilo D. (2010). Twin motion faster than the speed of sound. Phys. Rev. Lett..

[47] Kakeshita T., Kuroiwa K., Shimizu K., Ikeda T., Yamagishi A., Date M. (1993). Effect of Magnetic Fields on Athermal and Isothermal Martensitic Transformations in Fe-Ni-Mn Alloys. Mater. Trans. JIM.

[48] Lee Y.-H., Todai M., Okuyama T., Fukuda T., Kakeshita T., Kainuma R. (2011). Isothermal nature of martensitic transformation in an Ni_45_Co_5_Mn_36.5_In_13.5_ magnetic shape memory alloy. Scr. Mater..

[49] Kustov S., Golovin I., Corró M.L., Cesari E. (2010). Isothermal martensitic transformation in metamagnetic shape memory alloys. J. Appl. Phys..

[50] Chen F., Tong Y.X., Tian B., Zheng Y.F., Liu Y. (2010). Time effect of martensitic transformation in Ni_43_Co_7_Mn_41_Sn_9_. Intermetallics.

[51] Ito W., Ito K., Umetsu R.Y., Kainuma R., Koyama K., Watanabe K., Fujita A., Oikawa K., Ishida K., Kanomata T. (2008). Kinetic arrest of martensitic transformation in the NiCoMnIn metamagnetic shape memory alloy. Appl. Phys. Lett..

[52] Lakhani A., Banerjee A., Chaddah P., Chen X., Ramanujan R.V. (2012). Magnetic glass in shape memory alloy: Ni_45_Co_5_Mn_38_Sn_12_. J. Phys. Condens. Matter.

[53] Song Y., Chen X., Dabade V., Shield T.W., James R.D. (2013). Enhanced reversibility and unusual microstructure of a phase-transforming material. Nature.

[54] Srivastava V., Chen X., James R.D. (2010). Hysteresis and unusual magnetic properties in the singular Heusler alloy Ni_45_Co_5_Mn_40_Sn_10_. Appl. Phys. Lett..

[55] Delville R., Schryvers D., Zhang Z., James R.D. (2009). Transmission electron microscopy investigation of microstructures in low-hysteresis alloys with special lattice parameters. Scr. Mater..

[56] Lei C.H., Li L.J., Shu Y.C., Li J.Y. (2010). Austenite-martensite interface in shape memory alloys. Appl. Phys. Lett..

[57] Gebhardt T., Music D., Takahashi T., Schneider J.M. (2012). Combinatorial thin film materials science: From alloy discovery and optimization to alloy design. Thin Solid Films.

[58] Yang S., Wang C., Liu X. (2012). Phase equilibria and composition dependence of martensitic transformation in Ni-Mn-Ga ternary system. Intermetallics.

[59] Miyamoto T., Nagasako M., Kainuma R. (2013). Phase equilibria in the Ni–Mn–In alloy system. J. Alloy. Compd..

[60] Porthun S., ten Berge P., Lodder J.C. (1993). Bitter colloid observations of magnetic structures in perpendicular magnetic recording media. J. Magn. Magn. Mater..

[61] Ao W.Q., Liu F.S., Li J.Q., Du Y., Liu F.L. (2015). Isothermal section of the Ni–Mn–In ternary system at 773 K. J. Alloy. Compd..

[62] Wachtel E., Henninger F., Predel B. (1983). Constitution and magnetic properties of Ni-Mn-Sn alloys-solid and liquid state. J. Magn. Magn. Mater..

[63] Graf T., Felser C., Parkin S.S.P. (2011). Simple rules for the understanding of Heusler compounds. Prog. Solid State Chem..

[64] Chen F., Tong Y.X., Li L., Sánchez Llamazares J.L., Sánchez-Valdés C.F., Müllner P. (2017). The effect of step-like martensitic transformation on the magnetic entropy change of Ni_40.6_Co_8.5_Mn_40.9_Sn_10_ unidirectional crystal grown with the Bridgman-Stockbarger technique. J. Alloy. Compd..

[65] Laudise R.A., Sunder W.A., O’Bryan H.M., Carlson D.J., Witt A.F. (1992). Czochralski growth of single crystals of Ni_3−x_MnxSn. J. Cryst. Growth.

[66] Ito K., Ito W., Umetsu R.Y., Nagasako M., Kainuma R., Fujita A., Oikawa K., Ishida K. (2008). Martensitic transformation in NiCoMnSn metamagnetic shape memory alloy powders. Mater. Trans..

[67] Ahamed R., Ghomashchi R., Xie Z., Chen L., Munroe P., Xu S. (2018). Powder processing and characterisation of a quinary Ni-Mn-Co-Sn-Cu Heusler alloy. Powder Technol..

[68] Ito K., Ito W., Umetsu R.Y., Karaman I., Ishida K., Kainuma R. (2010). Mechanical and shape memory properties of Ni_43_Co_7_Mn_39_Sn_11_ alloy compacts fabricated by pressureless sintering. Scr. Mater..

[69] Ito K., Ito W., Umetsu R.Y., Karaman I., Ishida K., Kainuma R. (2011). Metamagnetic shape memory effect in Porous Ni_43_Co_7_Mn_39_Sn_11_ alloy compacts fabricated by pressureless sintering. Mater. Trans..

[70] Ito K., Ito W., Umetsu R.Y., Tajima S., Kawaura H., Kainuma R., Ishida K. (2009). Metamagnetic shape memory effect in polycrystalline NiCoMnSn alloy fabricated by spark plasma sintering. Scr. Mater..

[71] Monroe J.A., Cruz-Perez J., Yegin C., Karaman I., Geltmacher A.B., Everett R.K., Kainuma R. (2012). Magnetic response of porous NiCoMnSn metamagnetic shape memory alloys fabricated using solid-state replication. Scr. Mater..

[72] Anandh Vacuum Induction Melting Unit. http://home.iitk.ac.in/~anandh/lab/Induction%20Melting%20Unit2.pdf.

[73] Pérez-Sierra A.M., Pons J., Santamarta R., Vermaut P., Ochin P. (2015). Solidification process and effect of thermal treatments on Ni–Co–Mn–Sn metamagnetic shape memory alloys. Acta Mater..

[74] Yuhasz W.M., Schlagel D.L., Xing Q., Dennis K.W., McCallum R.W., Lograsso T.A. (2009). Influence of annealing and phase decomposition on the magnetostructural transitions in Ni_50_Mn_39_Sn_11_. J. Appl. Phys..

[75] Murakami Y., Watanabe Y., Kanaizuka T., Kachi S. (1981). Magnetic Properties and Phase Change of Ni_3-y_Mn_y_Sn Alloy. Trans. Jpn Inst. Metals.

[76] Schlagel D.L., McCallum R.W., Lograsso T.A. (2008). Influence of solidification microstructure on the magnetic properties of Ni-Mn-Sn Heusler alloys. J. Alloy. Compd..

[77] Bridgman-Stockbarger Technique. https://en.wikipedia.org/wiki/Bridgman%E2%80%93Stockbarger_technique.

[78] Huang L., Cong D.Y., Suo H.L., Wang Y.D. (2014). Giant magnetic refrigeration capacity near room temperature in Ni 40Co10Mn40Sn10 multifunctional alloy. Appl. Phys. Lett..

[79] Provenzano V., Shapiro A.J., Shull R.D. (2004). Reduction of hysteresis losses in the magnetic refrigerant Gd_5_Ge_2_Si_2_ by the addition of iron. Nature.

[80] Phan T.-L., Zhang P., Dan N.H., Yen N.H., Thanh P.T., Thanh T.D., Phan M.H., Yu S.C. (2012). Coexistence of conventional and inverse magnetocaloric effects and critical behaviors in Ni_50_Mn_50−x_Sn_x_ (x = 13 and 14) alloy ribbons. Appl. Phys. Lett..

[81] Huang Y.J., Liu J., Hu Q.D., Liu Q.H., Karaman I., Li J.G. Applications of the directional solidification in magnetic shape memory alloys. Proceedings of the 4th International Conference on Advances in Solidification Processes.

[82] Cong D.Y., Wang Y.D., Zetterstrom P., Peng R.L., Delaplane R., Zhao X., Zuo L. (2005). Crystal Structures and Textures of Hot Forged Ni_48_Mn_30_Ga_22_Alloy. Investigated by Neutron Diffraction Technique.

[83] Cong D.Y., Wang Y.D., Lin Peng R., Zetterstrom P., Zhao X., Liaw P.K., Zuo L. (2006). Crystal structures and textures in the hot-forged Ni-Mn-Ga shape memory alloys. Metall. Mater. Trans. A Phys. Met. Mater. Sci..

[84] Ma Y., Yang S., Liu Y., Liu X. (2009). The ductility and shape-memory properties of Ni-Mn-Co-Ga high-temperature shape-memory alloys. Acta Mater..

[85] Larin V.S., Torcunov A.V., Zhukov A., González J., Vazquez M., Panina L. (2002). Preparation and properties of glass-coated microwires. J. Magn. Magn. Mater..

[86] Rodionova V., Ilyn M., Granovsky A., Perov N., Zhukova V., Abrosimova G., Aronin A., Kiselev A., Zhukov A. (2013). Internal stress induced texture in Ni-Mn-Ga based glass-covered microwires. J. Appl. Phys..

[87] Zhukov A., Rodionova V., Ilyn M., Aliev A.M., Varga R., Michalik S., Aronin A., Abrosimova G., Kiselev A., Ipatov M. (2013). Magnetic properties and magnetocaloric effect in Heusler-type glass-coated NiMnGa microwires. J. Alloy. Compd..

[88] Shevyrtalov S., Zhukov A., Lyatun I., Medvedeva S., Miki H., Zhukova V., Rodionova V. (2018). Martensitic transformation behavior of Ni_2.44_Mn_0.48_Ga_1.08_ thin glass-coated microwire. J. Alloy. Compd..

[89] Shevyrtalov S., Zhukov A., Zhukova V., Rodionova V. (2018). Internal stresses influence on magnetic properties of Ni-Mn-Ga Heusler-type microwires. Intermetallics.

[90] Shevyrtalov S., Zhukov A., Medvedeva S., Lyatun I., Zhukova V., Rodionova V. (2018). Radial elemental and phase separation in Ni-Mn-Ga glass-coated microwires. J. Appl. Phys..

[91] Suryanarayana C., Veyssière P., Buschow K.H.J., Cahn R.W., Flemings M.C., Ilschner B., Kramer E.J., Mahajan S. (2002). Rapid Solidification Processing. Encyclopedia of Materials: Science and Technology.

[92] Sarı U., Aksoy İ. (2006). Electron microscopy study of 2H and 18R martensites in Cu–11.92 wt % Al–3.78 wt % Ni shape memory alloy. J. Alloy. Compd..

[93] Sade M., Lovey F.C. (1983). The structure of the modified 2H martensite in Cu-Zn-Al. Scr. Metall..

[94] Wang W., Li H., Ren J., Fu J., Zhai Q., Luo Z., Zheng H. (2015). Enhanced magnetocaloric properties in annealed Heusler Ni-Mn-Sn ribbons. J. Magn. Magn. Mater..

[95] Caballero-Flores R., González-Legarreta L., Rosa W.O., Sánchez T., Prida V.M., Escoda L., Suñol J.J., Batdalov A.B., Aliev A.M., Koledov V.V. (2015). Magnetocaloric effect, magnetostructural and magnetic phase transformations in Ni_50.3_Mn_36.5_Sn_13.2_ Heusler alloy ribbons. J. Alloy. Compd..

[96] Bruno N.M., Yegin C., Karaman I., Chen J.-H., Ross J.H., Liu J., Li J. (2014). The effect of heat treatments on Ni_43_Mn_42_Co_4_Sn_11_ meta-magnetic shape memory alloys for magnetic refrigeration. Acta Mater..

[97] Rama Rao N.V., Gopalan R., Manivel Raja M., Arout Chelvane J., Majumdar B., Chandrasekaran V. (2007). Magneto-structural transformation studies in melt-spun Ni-Mn-Ga ribbons. Scr. Mater..

[98] Hernando B., Llamazares J.L.S., Santos J.D., Sanchez M.L., Escoda L., Sunol J.J., Varga R., Garcia C., Gonzalez J. (2009). Grain oriented NiMnSn and NiMnIn Heusler alloys ribbons produced by melt spinning: Martensitic transformation and magnetic properties. J. Magn. Magn. Mater..

[99] Ma S.C., Shih C.W., Liu J., Yuan J.H., Lee S.Y., Lee Y.I., Chang H.W., Chang W.C. (2015). Wheel speed-dependent martensitic transformation and magnetocaloric effect in Ni-Co-Mn-Sn ferromagnetic shape memory alloy ribbons. Acta Mater..

[100] Chen X., Naik V.B., Mahendiran R., Ramanujan R.V. (2014). Optimization of Ni-Co-Mn-Sn Heusler alloy composition for near room temperature magnetic cooling. J. Alloy. Compd..

[101] Chen F., Tong Y.X., Huang Y.J., Tian B., Li L., Zheng Y.F. (2013). Suppression of gamma phase in Ni_38_Co_12_Mn _41_Sn_9_ alloy by melt spinning and its effect on martensitic transformation and magnetic properties. Intermetallics.

[102] Pandey S., Quetz A., Ibarra-Gaytan P.J., Sanchez-Valdes C.F., Aryal A., Dubenko I., Mazumdar D., Sanchez Llamazares J.L., Stadler S., Ali N. (2018). Effects of annealing on the magnetic properties and magnetocaloric effects of B doped Ni-Mn-In melt-spun ribbons. J. Alloy. Compd..

[103] Vajpai S.K., Dube R.K., Chatterjee P., Sangal S. (2012). A novel powder metallurgy processing approach to prepare fine-grained Cu-Al-Ni shape-memory alloy strips from elemental powders. Metall. Mater. Trans. A Phys. Met. Mater. Sci..

[104] Perez-Saez R.B., Recarte V., No M.L., Ruano O.A., San J.J. (2000). Advanced shape memory alloys processed by powder metallurgy. Adv. Eng. Mater..

[105] Monastyrsky G.E., Odnosum V., Van Humbeeck J., Kolomytsev V.I., Koval Y.N. (2002). Powder metallurgical processing of Ni–Ti–Zr alloys undergoing martensitic transformation: Part I. Intermetallics.

[106] Monastyrsky G.E., Van Humbeeck J., Kolomytsev V.I., Koval Y.N. (2002). Powder metallurgical processing of Ni–Ti–Zr alloys undergoing martensitic transformation—Part II. Intermetallics.

[107] Valeanu M., Lucaci M., Crisan A.D., Sofronie M., Leonat L., Kuncser V. (2011). Martensitic transformation of Ti_50_Ni_30_Cu_20_ alloy prepared by powder metallurgy. J. Alloy. Compd..

[108] Terayama A., Kyogoku H. (2010). Shape memory characteristics of the P/M-processed Ti–Ni–Cu alloys. Mater. Sci. Eng. A.

[109] Ibarra A., Rodriguez P.P., Recarte V., Perez-Landazabal J.I., No M.L., San Juan J. (2004). Internal friction behaviour during martensitic transformation in shape memory alloys processed by powder metallurgy. Mater. Sci. Eng. A.

[110] Kim Y.-W., Chung Y.-S., Choi E., Nam T.-H. (2012). Microstructure and Shape Memory Characteristics of Powder-Metallurgical-Processed Ti-Ni-Cu Alloys.

[111] Bertheville B. (2005). Powder metallurgical processing of ternary Ni_50_Ti_50−x_Zr_x_ (x = 5, 10 at %) alloys. J. Alloy. Compd..

[112] Zhang X., Xiang Y. (2017). Combinatorial approaches for high-throughput characterization of mechanical properties. J. Mater..

[113] Zarnetta R., Ehmann M., Savan A., Ludwig A. (2010). Identification of optimized Ti–Ni–Cu shape memory alloy compositions for high-frequency thin film microactuator applications. Smart Mater. Struct..

[114] Zarnetta R., Savan A., Thienhaus S., Ludwig A. (2007). Combinatorial study of phase transformation characteristics of a Ti–Ni–Pd shape memory thin film composition spread in view of microactuator applications. Appl. Surf. Sci..

[115] Cui J., Chu Y.S., Famodu O.O., Furuya Y., Hattrick-Simpers J., James R.D., Ludwig A., Thienhaus S., Wuttig M., Zhang Z. (2006). Combinatorial search of thermoelastic shape-memory alloys with extremely small hysteresis width. Nat. Mater..

[116] Zarnetta R., Takahashi R., Young M.L., Savan A., Furuya Y., Thienhaus S., Maa B., Rahim M., Frenzel J., Brunken H. (2010). Identification of quaternary shape memory alloys with near-zero thermal hysteresis and unprecedented functional stability. Adv. Funct. Mater..

[117] Takeuchi I., Famodu O.O., Read J.C., Aronova M.A., Chang K.S., Craciunescu C., Lofland S.E., Wuttig M., Wellstood F.C., Knauss L. (2003). Identification of novel compositions of ferromagnetic shape-memory alloys using composition spreads. Nat. Mater..

[118] Famodu O.O., Hattrick-Simpers J., Aronova M., Chang K.-S., Murakami M., Wuttig M., Okazaki T., Furuya Y., Knauss L.A., Bendersky L.A. (2004). Combinatorial Investigation of Ferromagnetic Shape-Memory Alloys in the Ni-Mn-Al Ternary System Using a Composition Spread Technique. Mater. Trans..

[119] Dwivedi A., Wyrobek T.J., Warren O.L., Hattrick-Simpers J., Famodu O.O., Takeuchi I. (2008). High-throughput screening of shape memory alloy thin-film spreads using nanoindentation. J. Appl. Phys..

[120] Liu Z., Wu Z., Yang H., Liu Y., Wang W., Ma X., Wu G. (2011). Martensitic transformation and magnetic properties in ferromagnetic shape memory alloy Ni_43_Mn_46_Sn_11−x_Six. Intermetallics.

[121] Aksoy S., Acet M., Wassermann E.F., Krenke T., Moya X., Manosa L., Planes A.P., Deen P. (2009). Structural properties and magnetic interactions in martensitic Ni-Mn-Sb alloys. Magazin.

[122] Dubenko I., Quetz A., Pandey S., Aryal A., Eubank M., Rodionov I., Prudnikov V., Granovsky A., Lahderanta E., Samanta T. (2015). Multifunctional properties related to magnetostructural transitions in ternary and quaternary Heusler alloys. J. Magn. Magn. Mater..

[123] Krenke T., Duman E., Acet M., Wasserman E.F., Moya X., Manosa L., Planes A., Suard E., Ouladdiaf B. (2007). Magnetic superelasticity and inverse magnetocaloric effect in Ni-Mn-In. Phys. Rev. B.

[124] Krenke T., Duman E., Acet M., Moya X., Manosa L., Planes A. (2007). Effect of Co and Fe on the inverse magnetocaloric properties of Ni-Mn-Sn. J. Appl. Phys..

[125] Wu Z., Liu Z., Yang H., Liu Y., Wu G. (2011). Effect of Co addition on martensitic phase transformation and magnetic properties of Mn_50_Ni_40_−xIn_10_Cox polycrystalline alloys. Intermetallics.

[126] Wang Z.L., Cong D.Y., Nie Z.H., Gao J., Liu W., Wang Y.D. (2012). The suppression and recovery of martensitic transformation in a Ni–Co–Mn–In magnetic shape memory alloy. J. Alloy. Compd..

[127] Pérez-Landazábal J.I., Lambri O.A., Bonifacich F.G., Sánchez-Alarcos V., Recarte V., Tarditti F. (2015). Influence of defects on the irreversible phase transition in Fe–Pd ferromagnetic shape memory alloys. Acta Mater..

[128] Meng Q., Yang H., Liu Y., Nam T.-H., Chen F. (2011). Thermal arrest analysis of thermoelastic martensitic transformations in shape memory alloys. J. Mater. Res..

[129] Brandon D., Kaplan W.D. (2008). Optical Microscopy. Microstructural Characterization of Materials.

[130] Krenke T., Acet M., Wasserman E.F., Moya X., Manosa L., Planes A. (2005). Martensitic transitions and nature of ferromagnetism in the austenitic and martensitic states of Ni-Mn-Sn alloys. Phys. Rev. B.

[131] Xu X., Ito W., Katakura I., Tokunaga M., Kainuma R. (2011). In situ optical microscopic observation of NiCoMnIn metamagnetic shape memory alloy under pulsed high magnetic field. Scr. Mater..

[132] Katakura I., Tokunaga M., Matsuo A., Kawaguchi K., Kindo K., Hitomi M., Akahoshi D., Kuwahara H. (2010). Development of high-speed polarizing imaging system for operation in high pulsed magnetic field. Rev. Sci. Instrum..

[133] Brandon D., Kaplan W.D. (2008). Diffraction Analysis of Crystal Structure. Microstructural Characterization of Materials.

[134] Bhatti K.P., El-Khatib S., Srivastava V., James R.D., Leighton C. (2012). Small-angle neutron scattering study of magnetic ordering and inhomogeneity across the martensitic phase transformation in Ni_50−x_Co_x_Mn_40_Sn_10_ alloys. Phys. Rev. B.

[135] Brown P.J., Crangle J., Kanomata T., Matsumoto M., Neumann K.U., Ouladdiaf B., Ziebeck K.R.A. (2002). The crystal structure and phase transitions of the magnetic shape memory compound Ni_2_MnGa. J. Phys. Condens. Matter.

[136] Brandon D., Kaplan W.D. (2008). Scanning Electron Microscopy. Microstructural Characterization of Materials.

[137] Brandon D., Kaplan W.D. (2008). Transmission Electron Microscopy. Microstructural Characterization of Materials.

[138] Delville R., Kasinathan S., Zhang Z., Humbeeck J.V., James R.D., Schryvers D. (2010). Transmission electron microscopy study of phase compatibility in low hysteresis shape memory alloys. Philos. Mag..

[139] Murakami Y., Yano T., Shindo D., Kainuma R., Arima T. (2007). Transmission Electron Microscopy on Magnetic Phase Transformations in Functional Materials.

[140] Hurrich C., Roth S., Wendrock H., Potschke M., Cong D.Y., Rellinghaus B., Schultz L. (2011). Influence of grain size and training temperature on strain of polycrystalline Ni_50_Mn_29_Ga_21_ samples. Proceedings of the Joint European Magnetic Symposia.

[141] Cong D.Y., Zhang Y.D., Esling C., Wang Y.D., Zhao X., Zuo L. (2008). Crystallographic features during martensitic transformation in Ni-Mn-Ga ferromagnetic shape memory alloys. Proceedings of the Materials Processing and Texture—15th International Conference on Textures of Materials.

[142] Yan H., Zhang Y., Xu N., Senyshyn A., Brokmeier H.-G., Esling C., Zhao X., Zuo L. (2015). Crystal structure determination of incommensurate modulated martensite in Ni–Mn–In Heusler alloys. Acta Mater..

[143] National SQUID Facility IIT Delhi What Is Superconducting Quantum Interface Device (SQUID)?. http://squid.iitd.ernet.in/Basic_Literature.htm.

[144] Shevyrtalov S., Miki H., Ohtsuka M., Grunin A., Lyatun I., Mashirov A., Seredina M., Khovaylo V., Rodionova V. (2018). Martensitic transformation in polycrystalline substrate-constrained and freestanding Ni-Mn-Ga films with Ni and Ga excess. J. Alloy. Compd..

[145] Lázpita P., Sasmaz M., Cesari E., Barandiarán J.M., Gutiérrez J., Chernenko V.A. (2016). Martensitic transformation and magnetic field induced effects in Ni_42_Co_8_Mn_39_Sn_11_ metamagnetic shape memory alloy. Acta Mater..

[146] Huang L., Cong D.Y., Ma L., Nie Z.H., Wang M.G., Wang Z.L., Suo H.L., Ren Y., Wang Y.D. (2015). Large magnetic entropy change and magnetoresistance in a Ni_41_Co_9_Mn_40_Sn_10_ magnetic shape memory alloy. J. Alloy. Compd..

[147] Zhang X., Qian M., Su R., Geng L. (2016). Giant room-temperature inverse and conventional magnetocaloric effects in Ni–Mn–In alloys. Mater. Lett..

[148] Zhang X., Qian M., Miao S., Su R., Liu Y., Geng L., Sun J. (2016). Enhanced magnetic entropy change and working temperature interval in Ni–Mn–In–Co alloys. J. Alloy. Compd..

[149] Sozinov A., Likhachev A.A., Lanska N., Soderberg O., Ullakko K., Lindroos V.K. (2003). Effect of Crystal Structure on Magnetic-Field-Induced Strain in Ni-Mn-Ga.

[150] Sozinov A., Lanska N., Soroka A., Zou W. (2013). 12% magnetic field-induced strain in Ni-Mn-Ga-based non-modulated martensite. Appl. Phys. Lett..

[151] Khalid F.A., Abbas S.Z. (2011). Characterization and properties of ferromagnetic shape memory alloys. Mater. Charact..

[152] Monroe J.A., Karaman I., Basaran B., Ito W., Umetsu R.Y., Kainuma R., Koyama K., Chumlyakov Y.I. (2012). Direct measurement of large reversible magnetic-field-induced strain in Ni-Co-Mn-In metamagnetic shape memory alloys. Acta Mater..

[153] Karaca H.E., Karaman I., Basaran B., Ren Y., Chumlyakov Y.I., Maier H.J. (2009). Magnetic field-induced phase transformation in NiMnColn magnetic shape-memory alloys-a new actuation mechanism with large work output. Adv. Funct. Mater..

[154] Li Z., Xu K., Zhang Y.L., Jing C. (2015). Reproducible magnetostrain behavior induced by structure transformation for Ni46Co4Mn39Sn11 Heusler alloy. J. Appl. Phys..

[155] He W.Q., Huang H.B., Liu Z.H., Ma X.Q. (2017). First-principles investigation of magnetic properties and metamagnetic transition of NiCoMnZ(Z = In, Sn, Sb) Heusler alloys. Intermetallics.

[156] Aguilar-Ortiz C.O., Soto-Parra D., Álvarez-Alonso P., Lázpita P., Salazar D., Castillo-Villa P.O., Flores-Zúñiga H., Chernenko V.A. (2016). Influence of Fe doping and magnetic field on martensitic transition in Ni–Mn–Sn melt-spun ribbons. Acta Mater..

[157] Yang L.H., Zhang H., Hu F.X., Sun J.R., Pan L.Q., Shen B.G. (2014). Magnetocaloric effect and martensitic transition in Ni_50_Mn_36−x_Co_x_Sn_14_. J. Alloy. Compd..

[158] Ingale B., Gopalan R., Raja M.M., Chandrasekaran V., Ram S. (2007). Magnetostructural transformation, microstructure, and magnetocaloric effect in Ni-Mn-Ga Heusler alloys. J. Appl. Phys..

[159] Rao N.V.R., Gopalan R., Chandrasekaran V., Suresh K.G. (2009). Microstructure, magnetic properties and magnetocaloric effect in melt-spun Ni-Mn-Ga ribbons. J. Alloy. Compd..

[160] Rama Rao N.V., Gopalan R., Chandrasekaran V., Suresh K.G. (2009). Phase coexistence, microstructure and magnetism in Ni-Mn-Sb alloys. J. Phys. D Appl. Phys..

[161] Zheng H., Wang W., Yu J., Zhai Q., Luo Z. (2014). Martensitic transformation in melt-spun Heusler Ni-Mn-Sn-Co ribbons. J. Mater. Res..

[162] Zheng H., Wang W., Xue S., Zhai Q., Frenzel J., Luo Z. (2013). Composition-dependent crystal structure and martensitic transformation in Heusler Ni-Mn-Sn alloys. Acta Mater..

[163] Gschneidner Jr K.A., Mudryk Y., Pecharsky V.K. (2012). On the nature of the magnetocaloric effect of the first-order magnetostructural transition. Scr. Mater..

[164] Trung N.T., Zhang L., Caron L., Buschow K.H.J., Brück E. (2010). Giant magnetocaloric effects by tailoring the phase transitions. Appl. Phys. Lett..

[165] Pérez-Sierra A.M., Bruno N.M., Pons J., Cesari E., Karaman I. (2016). Atomic order and martensitic transformation entropy change in Ni–Co–Mn–In metamagnetic shape memory alloys. Scr. Mater..

[166] Kustov S., Corró M.L., Pons J., Cesari E. (2009). Entropy change and effect of magnetic field on martensitic transformation in a metamagnetic Ni–Co–Mn–In shape memory alloy. Appl. Phys. Lett..

[167] Recarte V., Pérez-Landazábal J.I., Sánchez-Alarcos V., Rodríguez-Velamazán J.A. (2012). Dependence of the martensitic transformation and magnetic transition on the atomic order in Ni–Mn–In metamagnetic shape memory alloys. Acta Mater..

[168] Sánchez-Alarcos V., Recarte V., Pérez-Landazábal J.I., Gómez-Polo C., Rodríguez-Velamazán J.A. (2012). Role of magnetism on the martensitic transformation in Ni–Mn-based magnetic shape memory alloys. Acta Mater..

[169] Sánchez-Alarcos V., Recarte V., Pérez-Landazábal J., Cesari E., Rodríguez-Velamazán J. (2014). Long-Range Atomic Order and Entropy Change at the Martensitic Transformation in a Ni-Mn-In-Co Metamagnetic Shape Memory Alloy. Entropy.

[170] Yang S., Wang C., Shi Z., Wang J., Zhang J., Huang Y., Liu X. (2016). Microstructure, martensitic transformation, mechanical and shape memory properties of Ni–Co–Mn–In high-temperature shape memory alloys under different heat treatments. Mater. Sci. Eng. A.

[171] Ma Y., Jiang C., Li Y., Xu H., Wang C., Liu X. (2007). Study of Ni_50+x_Mn_25_Ga_25−x_ (x = 2–11) as high-temperature shape-memory alloys. Acta Mater..

[172] Yang S., Su Y., Wang C., Zhu J., Liu X. (2013). Microstructure, martensitic transformation and shape memory effect of Ni_38_Co_12_Mn_41_In_9_ alloy. Mater. Lett..

[173] Tan C., Zhang K., Tian X., Cai W. (2017). Effect of Gd addition on microstructure, martensitic transformation and mechanical properties of Ni_50_Mn_36_Sn_14_ ferromagnetic shape memory alloy. J. Alloy. Compd..

[174] Pérez-Checa A., Feuchtwanger J., Musiienko D., Sozinov A., Barandiaran J.M., Ullakko K., Chernenko V.A. (2017). High temperature Ni_45_Co_5_Mn_25−x_Fe_x_Ga_20_Cu_5_ ferromagnetic shape memory alloys. Scr. Mater..

[175] Zhao X.G., Hsieh C.C., Lai J.H., Cheng X.J., Chang W.C., Cui W.B., Liu W., Zhang Z.D. (2010). Effects of annealing on the magnetic entropy change and exchange bias behavior in melt-spun Ni–Mn–In ribbons. Scr. Mater..

[176] Yuhasz W.M., Schlagel D.L., Xing Q., McCallum R.W., Lograsso T.A. (2010). Metastability of ferromagnetic Ni–Mn–Sn Heusler alloys. J. Alloy. Compd..

[177] Das R., Saravanan P., Arvindha Babu D., Perumal A., Srinivasan A. (2013). Influence of solidification rate and heat treatment on magnetic refrigerant properties of melt spun Ni51Mn34In14Si1 ribbons. J. Magn. Magn. Mater..

[178] Sánchez-Alarcos V., Pérez-Landazábal J.I., Recarte V., Lucia I., Vélez J., Rodríguez-Velamazán J.A. (2013). Effect of high-temperature quenching on the magnetostructural transformations and the long-range atomic order of Ni–Mn–Sn and Ni–Mn–Sb metamagnetic shape memory alloys. Acta Mater..

